# Supplementary material for: Impact of COPD case finding on clinical care: a prospective analysis of the TargetCOPD trial
Source: BMJ Open. 2020 Oct 5;10(10):e038286. doi: 10.1136/bmjopen-2020-038286 (PMC7537458; doi:10.1136/bmjopen-2020-038286)
Supplement: Supplementary data [file bmjopen-2020-038286supp001.pdf]

Supplementary Table 1 Clinical codes used to extract data on COPD-related care from GP electronic health records

**Variable**	**Variable (specific)**	**Read code v2**	**Read code v3**
Antibiotic rescue pack		"8IEZ","8BMW"	"XaW9D","XaZp7"
Anxiety screening		"6897","388N"	"Xab9E","XaIwe"
Body mass index		"22K%"	"22K%"
COPD Care plan		"8CMV","66YI"	"8CMV","XaIUt"
Chest x-ray		"536"	"XE2az"
Clinical COPD Questionnaire (CCQ)		"38Dd"	"XaR6n"
COPD Assessment Test (CAT)		"38Dg"	"XaRFy"
COPD exception reporting		"9h5%"	"XaJ4R","XaJ4l","XaJ4k"
COPD review		"66YM"	"XaIet","XaXCb","XaXCa"
COPD severity		"H39","H36","H37","H38"	"XaEIV","XaN4a","XaEIY","XaEIW"
Depression screening		"6896%","6891%","388P","388w","E204","3885","38G5","388f"	"6891%","XaIwf","XaNkT","62T1","XaYS4","XaLDN"
Influenza vaccine		"65E%","n47%","9OX5"	"65E%","n47%","XaIBI"
Inhaler technique assessed/status		"6636","663I","663H","6637","66Y4"	"6636","663I","663H","6637","XaIQ0"
Prednisolone		"fe6%"	"fe6%"
Beclometasone dipropionate		"c61%","c66%"	"c61%","c66%"
Budesonide		"c64%"	"c64%"
Budesonide + formoterol (Symbicort)		"c67%"	"c67%"
Fluticasone + salmeterol (Seretide)		"c1D%"	"c1D%"
Fluticasone propionate		"c65%"	"c65%"
Indacaterol		"c1b%"	"c1b%"
Ipratropium bromide		"c31%"	"c31%"
Salbutamol		"c13%","c1E%"	"c13%","c1E%"
Salmeterol		"c19%"	"c19%"
Terbutaline		"c144","C145","C146","C147","c148","c14d","c14f","c14g","c14i","c14j","c14k","c14t","c14u","c14v","c14w","c14x"	"c144","C145","C146","C147","c148","c14d","c14f","c14g","c14i","c14j","c14k","c14t","c14u","c14v","c14w","c14x"
Aminophylline		"c41%"	"c41%"
Theophylline		"c43%"	"c43%"
MRC dyspnoea score		"173H","173I","173J","173K","173L"	"XaIUl","XaIUm","XaIUn","XaIUo","XaIUi"
Oxygen saturation		"44YA"	"X7708"
Oxygen therapy		"8771%"	"8771%","X71a%","XALL8"
Palliative care patients		"9EB5","8CM1%"	"XaJv2","XE2XB","XaCDx"

**Variable**	**Variable (specific)**	**Read code v2**	**Read code v3**
Physical activity level (GPPAQ)		"138b","138X","138a","138Y"	"XaPPE","XaPP8","XaPPD","XaPPB"
Pneumococcal vaccine		"6572","n4b%","8I3Q"	"6572","n4b%","XaIyy"
Pulmonary rehabilitation		"8H7u","8FA%","9NSL","8I86","8IA9"	"XaIf9","XaIUp","XaIUq","XS7qP","XaIUs" ,"XaZuM","XabGM","XaNQU"
Smoking cessation - medication	Bupropion / Nicotine replacement therapy / Varenicline	"du6%", "du3%","du7%", "du8%"	"du6%", "du3%","du7%", "du8%"
Smoking cessation	Declined smoking cessation support	"9NdZ"	"XaRFh"
Smoking cessation	Smoking cessation advice	"8CAL"	"Ua1Nz"
Smoking status	Current/ex/never	"137%"	"137%"
Spirometry	FEV1: actual / predicted / % predicted, FEV1/FVC ratio: actual / predicted / % predicted, FVC: actual / predicted / % predicted	"339S","339O","339P","339R","339M","339N","3396%","339Q"	"XaEFz","X77Qu","XaCJK","XaEFy","X77Ra","XaCFR","3396%","XaQmT","XaPpI","XaCJL","XaJ3K"
Spirometry		"745D4","8HRC"	"XaXeg","XaK02"

Supplementary Table 2 Clinical codes used to define a clinician diagnosis of COPD

Clinical system: EMIS Web and LV	
H31	Chronic bronchitis
H32	Emphysema
H36	Mild chronic obstructive pulmonary disease
H37	Moderate chronic obstructive pulmonary disease
H38	Severe chronic obstructive pulmonary disease
H39	Very severe chronic obstructive pulmonary disease
H3A	End stage chronic obstructive airways disease
H3y	Other specific chronic obstructive airways disease
H3z	Chronic obstructive airways disease
Clinical system: System One	
H3	COPD read codes in a cluster

Supplementary Table 3 Participant characteristics of those with and without additional electronic health record (EHR) or questionnaire data

**Participant characteristics**	**With additional EHR data**	**With follow-up questionnaire data**	**Without additional EHR or follow-up questionnaire data**
Mean age (SD)	63.8 (9.4)	64.7 (8.9)	63.2 (10.2)
% Male	62.2	58.7	54.2
% Current smoker	37.7	29.2	37.3
Mean FEV_1 % predicted (SD)	86.3 (18.1)	87.8 (17.9)	84.8 (18.4)

Supplementary Table 4 Participant characteristics of those with questionnaire data (n=375)

**Participant characteristics**		**N**	**(%)**
Median age (IQR)		65.8	(58.8-71.0)
Sex	Male	220	(58.7)
Smoking status	Current	77	(20.5)
	Former	202	(53.9)
	Never	41	(10.9)
FEV_1 % predicted	Mean	87.8	(17.9)
	≥80	249	(66.4)
	50-79	121	(32.3)
	<50	5	(1.3)
Comorbidities	Asthma	31	(8.3)
	Ischaemic heart disease	22	(5.9)
	Diabetes	20	(5.3)
	Depression	5	(1.3)
	Anxiety	6	(1.6)
CAT score (median, IQR)		12	(7-19)
Symptoms	MRC dyspnoea ≥3	148	(39.5)
	Chronic cough	128	(34.1)
	Phlegm	194	(51.7)
	Wheeze	274	(73.1)

CAT=COPD Assessment Test, FEV1=forced expiratory volume in 1 second

Supplementary Table 5 Clinical care of participants with electronic health record data who were newly diagnosed with COPD by case-finding and usual care*

		**Diagnostic approach**			
		**Case finding (n=341)**		**Usual care (n=188)**	
		**N****	**(%)**	**N****	**(%)**
Clinical assessment					
MRC score recorded		107	(31.4)	176	(93.6)
CAT score recorded		39	(11.4)	102	(54.3)
Spirometry undertaken		101	(29.6)	147	(78.2)
COPD severity recorded		48	(14.1)	118	(62.8)
BMI recorded		277	(81.2)	178	(94.7)
Oxygen saturations recorded		50	(14.7)	66	(35.1)
CXR undertaken		22	(6.5)	20	(10.6)
Depression screen undertaken		71	(20.8)	64	(34.0)
Clinical intervention					
Listed on COPD register		78	(22.9)	175	(93.1)
Care plan recorded		41	(12.0)	119	(63.3)
Annual review undertaken		95	(27.9)	176	(93.6)
Smoking cessation counselling provided		185	(54.3)	154	(81.9)
Nicotine replacement therapy		39	(11.4)	29	(15.4)
Influenza vaccination provided		245	(71.8)	144	(76.6)
Pneumococcal vaccine provided		29	(8.5)	28	(14.9)
Pulmonary rehabilitation provided		20	(5.9)	52	(27.7)
Inhaler technique assessed		73	(21.4)	140	(74.5)
Inhalers prescribed	Salbutamol	146	(42.8)	167	(88.8)
	Ipratropium	8	(2.3)	15	(8.0)
	Salmeterol	3	(0.9)	15	(8.0)
	Fluticasone	3	(0.9)	1	(0.5)
	Budesonide	1	(0.3)	0	(0.0)
	Beclometasone	26	(7.6)	23	(12.2)
	Fluticasone/salmeterol	37	(10.9)	77	(41.0)
	Budesonide/formoterol	12	(3.5)	26	(13.8)
	Any of the above inhalers	151	(44.3)	172	(91.5)
Antibiotic rescue pack		11	(3.2)	48	(25.5)
Prednisolone		69	(20.2)	114	(60.6)
**Clinical care score**	<5	225	(66.0)	17	(9.0)
	5 to 9	83	(24.3)	73	(38.8)
	≥10	33	(9.7)	98	(52.1)
	Median (IQR)	4	(2 to 6)	11	(9 to 13)

*Includes data from the start up to 2 years following the end of the TargetCOPD trial
**Number of participants who received the clinical assessment or intervention
BMI=body mass index, CAT=COPD Assessment Test, IQR=interquartile range, MRC=Medical Research Council

Supplementary Table 6 Multilevel linear regression model assessing the association between the clinical care score and participant characteristics, among those with electronic health record data (n=467)*

**Participant characteristics**	**aβ***	**(95% CI)**	**p**
Age	0.03	(0.00, 0.05)	0.022
Sex (male)	-0.35	(-0.80, 0.09)	0.121
Smoking status			
Ex-smoker	0.01	(-0.84, 0.86)	0.987
Current smoker	1.09	(0.21, 1.97)	0.015
No. of comorbidities	0.32	(0.09, 0.55)	0.006
Case-found vs. routinely diagnosed	-0.48	(-1.21, 0.24)	0.189
Listed on COPD register	6.15	(5.49, 6.82)	<0.001

* Includes data from the start up to 2 years following the end of the TargetCOPD trial
