# Supplementary material for: Impact of COPD case finding on clinical care: a prospective analysis of the TargetCOPD trial
Source: BMJ Open. 2020 Oct 5;10(10):e038286. doi: 10.1136/bmjopen-2020-038286 (PMC7537458; doi:10.1136/bmjopen-2020-038286)
Supplement: Author's manuscript [file bmjopen-2020-038286.draft_revisions.pdf]

BMJ Open

BMJ Open is committed to open peer review. As part of this commitment we make the peer review history of every article we publish publicly available.

When an article is published we post the peer reviewers' comments and the authors' responses online. We also post the versions of the paper that were used during peer review. These are the versions that the peer review comments apply to.

The versions of the paper that follow are the versions that were submitted during the peer review process. They are not the versions of record or the final published versions. They should not be cited or distributed as the published version of this manuscript.

BMJ Open is an open access journal and the full, final, typeset and author-corrected version of record of the manuscript is available on our site with no access controls, subscription charges or pay-per-view fees (http://bmjopen.bmj.com).

If you have any questions on BMJ Open's open peer review process please email info.bmjopen@bmj.com

BMJ Open

# The impact of COPD case finding on clinical care: a prospective analysis of the TargetCOPD trial

| | |
|---|---|
| Journal: | *BMJ Open* |
| Manuscript ID | bmjopen-2020-038286 |
| Article Type: | Original research |
| Date Submitted by the Author: | 05-Mar-2020 |
| Complete List of Authors: | Haroon, Shamil; University of Birmingham,<br>Adab, Peymane; University of Birmingham, Public Health<br>Dickens, Andy; University of Birmingham, Institute of Applied Health Research<br>Sitch, Alice; University of Birmingham, Public Health<br>Rai, Kiran; University of Birmingham, Institute of Applied Health Research<br>Enocson, Alexandra; University of Birmingham, Applied Health Research<br>Fitzmaurice, David; University of Warwick Warwick Medical School<br>Jordan, Rachel; University of Birmingham, Public Health and Epidemiology |
| Keywords: | Chronic airways disease < THORACIC MEDICINE, PRIMARY CARE, PUBLIC HEALTH |
| | |

SCHOLARONE™
Manuscripts

*I, the Submitting Author has the right to grant and does grant on behalf of all authors of the Work (as defined in the below author licence), an exclusive licence and/or a non-exclusive licence for contributions from authors who are: i) UK Crown employees; ii) where BMJ has agreed a CC-BY licence shall apply, and/or iii) in accordance with the terms applicable for US Federal Government officers or employees acting as part of their official duties; on a worldwide, perpetual, irrevocable, royalty-free basis to BMJ Publishing Group Ltd ("BMJ") its licensees and where the relevant Journal is co-owned by BMJ to the co-owners of the Journal, to publish the Work in this journal and any other BMJ products and to exploit all rights, as set out in our licence.*

*The Submitting Author accepts and understands that any supply made under these terms is made by BMJ to the Submitting Author unless you are acting as an employee on behalf of your employer or a postgraduate student of an affiliated institution which is paying any applicable article publishing charge ("APC") for Open Access articles. Where the Submitting Author wishes to make the Work available on an Open Access basis (and intends to pay the relevant APC), the terms of reuse of such Open Access shall be governed by a Creative Commons licence – details of these licences and which Creative Commons licence will apply to this Work are set out in our licence referred to above.*

*Other than as permitted in any relevant BMJ Author's Self Archiving Policies, I confirm this Work has not been accepted for publication elsewhere, is not being considered for publication elsewhere and does not duplicate material already published. I confirm all authors consent to publication of this Work and authorise the granting of this licence.*

For peer review only

# The impact of COPD case finding on clinical care: a prospective analysis of the TargetCOPD trial

Authors: Shamil Haroon* (PhD), Peymané Adab* (MD), Andrew P. Dickens* (PhD), Alice J. Sitch** (PhD), Kiran Rai* (PhD), Alexandra Enocson* (PhD), David A. Fitzmaurice*** (MD), and Rachel E. Jordan* (PhD)

*Institute of Applied Health Research, College of Medical and Dental Sciences, University of Birmingham, Edgbaston, Birmingham, B15 2TT, UK.

**NIHR Birmingham Biomedical Research Centre, University Hospitals Birmingham NHS Foundation Trust and University of Birmingham, UK.

***Warwick Medical School, University of Warwick, Coventry, CV4 7AL, UK.

Corresponding authors: Rachel Jordan (r.e.jordan@bham.ac.uk), T: +44 121 414 6775

Shamil Haroon (s.haroon@bham.ac.uk), T: +44 121 414 5042

Word count: Abstract- 274

Main text- 3610

Key words: COPD, case finding, screening, primary care, clinical management

For peer review only

# Abstract

## Objectives

To investigate the impact of COPD case finding on clinical care.

## Design

We conducted a prospective observational analysis of data from TargetCOPD, a pragmatic cluster RCT in primary care in the West Midlands, UK. This compared alternative methods of COPD case finding against usual care. Data were extracted from electronic healthcare records and self-reported questionnaires for a subset of patients with newly diagnosed COPD.

## Setting

50 general practices that participated in the TargetCOPD trial.

## Participants

Patients aged 40 to 79 years newly identified with COPD by targeted case finding or by usual care, from 10th August 2012 to 22nd June 2014.

## Primary and secondary outcome measures

The primary outcome was addition to a COPD register by the end of the trial. The secondary outcome was a clinical care score, derived from the sum of clinical assessments and relevant interventions. Associations between participant characteristics and the primary and secondary outcomes were assessed using multilevel regression.

## Results

857 patients identified with COPD by case finding and 764 by usual care were included. Only 21.2% of case-found patients had been added to a COPD register, compared to 92.7% of those diagnosed by usual care. The odds of being added were greater in smokers (adjusted odds ratio 8.68, 95% CI 2.53-29.8), and in those with lower percentage of predicted $FEV_1$ (adjusted OR 0.96 per percentage rise,




For peer review only - http://bmjopen.bmj.com/site/about/guidelines.xhtml

95% CI 0.95-0.98). Patients who had been added to a COPD register had a significantly higher clinical care score (mean difference 5.06, 95% CI 4.36-5.75).

## Conclusions

Only one in five case-found patients had been registered with COPD. Patients added to a COPD register received significantly higher levels of appropriate clinical care.

For peer review only



For peer review only - http://bmjopen.bmj.com/site/about/guidelines.xhtml

# Strengths and limitations of this study

- We conducted a detailed evaluation of the respiratory care received by patients with case-found COPD and compared this to that received by patients diagnosed by usual care over the same period.
- The analyses adjusted for a number of important confounding factors, including measures of disease severity.
- The analysis was done on only a subset of trial participants.
- Electronic health record data quality was highly dependent on clinical coding practices.
- The clinical care score represents a relatively crude estimate of overall respiratory care and individual components of the score were not weighted for their relative importance.

For peer review only



For peer review only - http://bmjopen.bmj.com/site/about/guidelines.xhtml

# Introduction

COPD is the third leading cause of death worldwide and a major cause of disability.[1] There is a large burden of undiagnosed COPD globally and a number of experts and policy makers have called for early detection and screening.[2] This has the potential to prevent a significant burden of premature morbidity and mortality. A targeted case finding approach in England could reduce COPD hospitalisations by an estimated 3300 per year and prevent almost 3000 premature deaths over 3 years.[3] A recent model-based evaluation also concluded that systematic case finding for COPD could be cost-effective in the long term.[4] However, this is based on the assumption that case-found patients go on to receive improved clinical care.

The UK National Screening Committee (NSC) requires evidence from high quality randomised controlled trials (RCTs) that a screening programme reduces mortality or morbidity and that the benefits outweigh any harms, before recommending population-based screening.[5] A systematic review for the US Preventive Services Task Force concluded that screening asymptomatic people for COPD could not be recommended because of a lack of evidence that it improves health-related quality of life, morbidity or mortality.[6] However, this does not necessarily apply to patients with symptoms.[7]

A large number of studies have evaluated case finding approaches for COPD in primary care.[8,9] However, few have assessed the clinical outcomes of case-found patients compared to those diagnosed through usual care. TargetCOPD was a large cluster RCT in the West Midlands, UK which confirmed the effectiveness and cost-effectiveness of a targeted programme to identify new COPD cases in the primary care setting compared to usual care.[10]

Follow-up data were collected on participants identified with COPD during the trial period to assess the clinical interventions they subsequently received. The objective of this study was to describe the clinical care and management of patients newly identified with COPD by targeted case finding and compare this with those diagnosed by usual care over the same period. In addition, we assessed which



For peer review only - http://bmjopen.bmj.com/site/about/guidelines.xhtml

patient and practice-level factors were associated with better management of COPD among those who were newly diagnosed.



For peer review only - http://bmjopen.bmj.com/site/about/guidelines.xhtml

# Methods

## Study design

We undertook a prospective observational analysis of data on the clinical care of patients newly diagnosed with COPD in the TargetCOPD trial.[10] TargetCOPD was a cluster RCT comparing the effectiveness and cost-effectiveness of alternative approaches to targeted case finding against usual care on the identification of previously undiagnosed COPD.

Patients aged 40-79 years with no prior diagnosis of COPD were identified from electronic general practice registers. Those in the case finding arm were provided screening questionnaires by post and/or opportunistically when visiting their practice. Patients reporting respiratory symptoms were then invited for a spirometry assessment. Post-bronchodilator spirometry was performed by trained researchers according to American Thoracic Society and European Respiratory Society 2005 guidelines[11] using an ultrasonic flow head spirometer (Spiroson-AS, ndd Medical Technologies, Zurich, Switzerland).

The study team sent letters to GPs informing them of patients identified as likely to have COPD through case-finding (see below for definition) with advice to follow the relevant National Institute for Health and Care Excellence (NICE) guidelines.[12] Patients newly diagnosed either by case finding or by usual care during the trial period were tracked through GP records. The trial was active in each practice for a period of one year, with a staggered start from 10th August 2012 to 22nd June 2013.

From 6th October 2015 to 12th October 2016, data on the clinical care of COPD were extracted from the electronic healthcare records (EHRs) of a sample of eligible patients from both arms of the trial (clinical codes listed in Supplementary Table 1). All case-found patients who agreed to further study were sent questionnaires from 30th March to 11th April 2018 that included detailed questions about the management of their COPD.



For peer review only - http://bmjopen.bmj.com/site/about/guidelines.xhtml

## Setting

This analysis used data from 50 out of 54 general practices that participated in the TargetCOPD trial. Relevant data could not be extracted from four practices due to practice closures (n=3) and missing practice lists (n=1).

## Participants

In this study we included those aged 40 to 79 years at baseline who were newly identified with COPD by targeted case finding or by usual care, from 10th August 2012 to 22nd June 2014. Case-found COPD was defined as a post-bronchodilator forced expiratory volume in one second to forced vital capacity ratio ($FEV_1$/FVC) of less than 0.7 (in line with recommended UK guidelines at the time) in those who reported respiratory symptoms (chronic cough or phlegm for at least three months of the year for two or more years, wheeze in the previous 12 months or MRC grade 2 dyspnoea or higher). Newly diagnosed COPD by usual care was defined as a clinician diagnosis recorded on the EHR using pre-defined clinical codes (Supplementary Table 2) made independently of case finding. This included all patients diagnosed with COPD in the usual care arm and those diagnosed in the case finding arm prior to receiving a trial spirometry assessment.

## Outcomes

The primary outcome was the addition of patients to a COPD register by the close of the trial period. There is currently a contractual requirement for general practices in England to maintain a register of patients with COPD.[13] Addition to a register is clinician-led but supported by administrators.

The secondary outcome was a composite clinical care score, derived by summing the number of clinical assessments and interventions recorded (one point for each) from the end of the trial up to two years of follow-up. The components of the score were based on clinical assessments and interventions that were relevant to this patient group, based on NICE guidelines.[12] Clinical assessments included recording MRC dyspnoea score, COPD assessment test (CAT), post-bronchodilator spirometry, COPD severity, body mass index, oxygen saturation, chest X-ray, and screening for depression and anxiety. Clinical interventions included recording a care plan, annual review, smoking cessation



For peer review only - http://bmjopen.bmj.com/site/about/guidelines.xhtml

support (brief advice and nicotine replacement therapy [NRT] for current smokers), influenza and pneumococcal vaccination, referral for pulmonary rehabilitation, assessment of inhaler technique, and prescriptions of inhalers, antibiotics, and prednisolone. Sensitivity analyses included data from the beginning of the trial up to three years of follow-up.

## Other variables

Data on demographic characteristics, smoking status, and comorbidities (asthma, ischaemic heart disease, heart failure, diabetes, depression, anxiety, osteoporosis, and stroke) were also obtained for included participants from the main trial dataset. Practice level data were extracted including patient list size, socioeconomic status (based on the Index of Multiple Deprivation [IMD]), percentage of the registered patient list from non-white ethnicities, and the baseline percentage of patients already diagnosed with COPD (data provided by each practice).

## Statistical methods

The primary analysis used a multilevel logistic regression model to investigate the association between the odds of being added to a COPD register and patient characteristics, among those with case-found COPD. This included the practice as a cluster variable, and demographic and clinical characteristics as independent variables, adjusting for age, sex, smoking status, percentage of predicted $FEV_1$, number of self-reported comorbidities, and CAT score. A complete case analysis was performed due to low levels of missing data.

The secondary analysis used a multilevel linear regression model among participants diagnosed with COPD during the trial to assess associations between the clinical care score and patient characteristics, diagnostic route (case-found vs. usual care), and addition to a COPD register. The model was run separately using EHR and questionnaire data. Associations between the percentage of case-found patients added to COPD registers and practice characteristics were explored using Pearson correlation coefficients. All analyses were performed using Stata M/P version 14.2.



For peer review only - http://bmjopen.bmj.com/site/about/guidelines.xhtml

## Ethical approval

The Solihull Research Ethics Committee provided ethical approval for the TargetCOPD trial (IRAS, reference 11/WM/0403).

## Role of the funding source

The study sponsors had no role in the study design; in the collection, analysis, and interpretation of data; in the writing of the report; and in the decision to submit the paper for publication. The corresponding authors had full access to all the data in the study and have final responsibility for the decision to submit for publication.

For peer review only



For peer review only - http://bmjopen.bmj.com/site/about/guidelines.xhtml

# Results

## Participants

Over the trial period, 1621 people with previously undiagnosed COPD were identified in participating practices, including 857 through case finding, and 764 by usual care (337 of these from the usual care arm).[14] EHR data on clinical care were available for 532 patients who were identified with COPD through either case finding or usual care (figure 1). Among the 857 case-found patients, 375 returned questionnaires. The characteristics of participants with and without additional EHR and questionnaire data were broadly similar (Supplementary Table 3).

The mean age of participants newly diagnosed with COPD by case finding and usual care was similar (63.8 and 63.7 years, respectively), a similar proportion were male, and the majority (65.6%) were white British (Table 1). A greater proportion of participants diagnosed by usual care were current smokers compared with those diagnosed by case finding (54.6% vs. 30.2%), although the proportion with chronic conditions was similar. The sub-sample with case-found COPD who returned a questionnaire were slightly older, more likely to be male, and generally reported fewer comorbidities than the full sample of case-found patients (Supplementary Table 4).

## Practice characteristics

The mean practice list size was 5762 patients (SD 3482). The mean socioeconomic status (mean IMD score 36.7, SD 15.2) was similar to Birmingham as a whole (mean 37.8).[15] The majority of registered patients were white British (mean 80.5%, SD 20.5) and the mean prevalence of diagnosed COPD at the start of the trial was 1.6% (SD 0.6).

## Addition to COPD registers

Of the 857 patients with case-found COPD, only 182 (21.2%) had been added to a COPD register, compared to 708 out of 764 patients (92.7%) diagnosed by usual care. Among those with case-found COPD, the median time from the trial spirometry assessment to being added to a COPD register was 152 days (IQR 72 to 258).



For peer review only - http://bmjopen.bmj.com/site/about/guidelines.xhtml

Among case-found patients, the odds of being added to a COPD register were higher among current or former smokers (adjusted odds ratio 8.68, 95% CI 2.53 to 29.8, and 6.32, 95% CI 1.88 to 21.3, respectively), and those with a lower percentage of predicted $FEV_1$ (adjusted OR 0.96 per percentage rise, 95% CI 0.95 to 0.98; Table 2).

The median percentage of case-found patients added to a COPD register in each general practice was 14.5% (IQR 3.2% to 32.3%). Overall there were no significant correlations between the percentage of patients added to a COPD register and the measured practice characteristics (Table 3).

## Clinical care of newly diagnosed COPD

Among patients with EHR data, clinical assessments that were commonly performed within the two year follow-up period included measurement of BMI (77.4%) and documentation of the MRC dyspnoea score (50.6%; Table 4). Documentation of CAT score, disease severity, oxygen saturation, chest X-ray, and depression screening, was infrequent. All aspects of clinical assessment were more commonly performed for patients diagnosed through usual care than for those by case finding.

Therapies that were commonly delivered included influenza vaccination (69.8% vs. 73.4% for those diagnosed through case finding and usual care, respectively), smoking cessation counselling for current smokers (45.6% vs. 73.9%), and prescription of inhalers (39.0% vs. 86.7%). Prescription of NRT, pulmonary rehabilitation, and pneumococcal vaccination were infrequent. The median clinical care score was significantly higher among participants who had been diagnosed by usual care than those by case finding (10 vs. 3, respectively).

Among case-found patients with questionnaire data (n=375), those added to a COPD register (n=78 [20.8%]) were more likely to have been informed of their COPD diagnosis (88.5% vs 17.5%; Table 5). They were also more likely to have received a number of clinical interventions, including a care plan (79.5% vs 13.1%), influenza and pneumococcal vaccination (85.9% vs 69.7%, and 55.1% vs 40.7%, respectively), and prescriptions of inhalers (74.4% vs 35.0%). Very few had been offered or referred to a pulmonary rehabilitation service irrespective of whether they had been added to a COPD register.



For peer review only - http://bmjopen.bmj.com/site/about/guidelines.xhtml

## Factors associated with higher levels of clinical care

Using EHR data, patients who had been added to a COPD register had a clinical care score 5 points higher than those who had not (adjusted mean difference 5.06, 95% CI 4.36 to 5.75; Table 6). This was also found to a lesser extent for those with a higher number of comorbidities (adjusted mean difference 0.38 per additional comorbidity, 95% CI 0.11 to 0.65). These findings remained consistent in sensitivity analyses when data from the beginning of the trial were also included, although current smoking also became significantly associated with an increase in the clinical care score (1.09, 95% CI 0.21 to 1.97; Supplementary Tables 5 and 6).

Similarly, among those with questionnaire data, patients who had been added to a COPD register had a higher clinical care score than those who had not (adjusted mean difference 3.48, 95% CI 2.81 to 4.15). This was also true to a lesser extent for those who had a higher CAT score (0.05 per unit rise in CAT score, 95% CI 0.01 to 0.08), and lower percentage of predicted $FEV_1$ (-0.02 per percentage rise, 95% CI -0.03 to -0.01; Table ).

For peer review only



For peer review only - http://bmjopen.bmj.com/site/about/guidelines.xhtml

# Discussion

## Main findings

Only one in five patients newly diagnosed with COPD through targeted case finding in primary care had been added to a COPD register by the close of the TargetCOPD trial, compared to more than 90% of those diagnosed by usual care. Addition to the register was more likely among current and former smokers, and those with poorer lung function. Practice characteristics did not correlate with the percentage of case-found patients added to COPD registers.

The clinical care of COPD was significantly more comprehensive for patients who had been diagnosed by usual care than those by case finding, receiving on average seven more clinical assessments or interventions. This was mainly because patients diagnosed by usual care were significantly more likely to be added to a COPD register. In addition, patients with a higher number of comorbidities, worse CAT scores, poorer lung function, and current smokers, were also more likely to receive a higher level of respiratory care.

Very few patients diagnosed by either approach had been offered or referred to pulmonary rehabilitation, irrespective of whether they had been added to a COPD register. Also, relatively few had been administered a pneumococcal vaccine or provided adequate smoking cessation support.

## Relationship to other studies

Only two published studies have evaluated the clinical care of patients newly diagnosed with COPD by case finding. Similar to our findings, these also showed that case-finding was not followed by adequate COPD management for most patients. One study based in the Netherlands examined community-dwelling frail patients aged 65 years and older with dyspnoea who had participated in a screening study for COPD and heart failure.[16] During six months of follow-up, only 13.7% (n=53) of the new cases of COPD had any changes made to respiratory drug prescriptions.

A large cluster RCT of COPD screening in the USA similarly found that respiratory-related clinical activity was limited following identification, with only 187 of 994 patients (19%) who screened



For peer review only - http://bmjopen.bmj.com/site/about/guidelines.xhtml

positive for COPD receiving a respiratory intervention.[17] The study examined a limited number of clinical activities, which included referral for pulmonary function testing, referral to a respiratory specialist, and new respiratory medication prescriptions. The likelihood of receiving this care was associated with prior visits for respiratory issues and previous prescriptions of respiratory medications.[18]

A qualitative study exploring the views of healthcare providers within the TargetCOPD trial on screening, suggested that poor knowledge, lower perceived priority, and insufficient resources for COPD diagnosis and management were barriers to adequate COPD management in primary care.[19] A qualitative study with patients also found that GPs often lack the time to engage in case finding, that accessing primary care appointments can be difficult, and that communication about a diagnosis can often be lacking.[20] In addition, patients may occasionally be in denial of their respiratory symptoms or may not prioritise this over other health complaints. Case finding strategies will therefore need to address patient education on accessing health services more promptly for respiratory symptoms.

A recent literature review of COPD management in primary care found that there is significant variability in the provision of recommended treatments, with barriers including a lack of familiarity with clinical guidelines.[21] This is reflected in prescribing practices. An analysis of UK primary care prescribing data in 2014 among 24,957 patients found that COPD is often not managed according to GOLD or NICE guidelines.[22] 18% of GOLD stage 2 patients had received no treatment despite having symptoms, and a significant proportion had received inhaled corticosteroids irrespective of their disease severity and exacerbation history. Similar findings of inconsistent prescribing were found in a study assessing the management of COPD in a primary care clinic in the USA.[23]

More recently, the national COPD primary care audit in Wales found significant shortcomings in the clinical management of patients with COPD.[24] Only 12.5% of smokers had received smoking cessation support, 34.0% had not received an influenza vaccination and half of patients with MRC dyspnoea grades 3-5 had not been referred for pulmonary rehabilitation. A study of COPD care in community pharmacies in Belgium similarly found low rates of influenza vaccination in patients younger than 65 years, poor medication adherence, and poor inhaler technique, among a significant



For peer review only - http://bmjopen.bmj.com/site/about/guidelines.xhtml

proportion of COPD patients.[25] Findings from the Continuing to Confront COPD Survey suggested that there are likely to be significant shortcomings in the provision of guideline-recommended treatments among both primary and secondary care clinicians internationally.[26]

The UK National Screening Committee recommend that clinical service provision and patient outcomes should be optimised in all healthcare providers prior to participation in a screening programme.[5] These widespread gaps in care provision will need to be addressed before recommendations for targeted case finding can be made.

## Limitations

Limitations include the unavailability of data on all trial participants. Patient questionnaires were only available for 375 case-found patients and EHR data on clinical care for a subset of participants (344 out of 857 [40.1%] diagnosed by case finding and 188 out of 764 [24.6%] by usual care). However, the demographic and clinical characteristics of patients with and without relevant data were broadly similar, suggesting that the findings are likely to apply to the full study population (Supplementary Table 3). There was also limited data on disease severity, particularly in the usual care arm of the TargetCOPD trial. However, our analyses adjusted for measures of disease severity such as percentage of predicted $FEV_1$, CAT score, and number of comorbidities.

EHR data quality was highly dependent on clinical coding by participating practices. These were routinely collected health service data and were not specifically recorded for research purposes. Inadequate recording could lead to an underestimation of the level of clinical care provided. Furthermore, a significant amount of smoking cessation support in England is provided in community pharmacies, which would not necessarily be captured in GP records. However, self-reported questionnaire data on smoking cessation support were also collected from a subset of patients, and had broadly similar findings.

Finally, the clinical care score represents a relatively crude estimate of overall respiratory care and individual components of the score were not weighted for their relative importance. Some of the components reflect the management of COPD exacerbations, which may have differed in their



For peer review only - http://bmjopen.bmj.com/site/about/guidelines.xhtml

incidence between case-found and routinely diagnosed patients. However, there are currently no validated methods or scores for quantifying overall levels of care for COPD and we therefore chose what we considered a reasonable and pragmatic approach.

## Implications for practice, policy and research

Our findings suggest that COPD case finding in primary care is unlikely to result in improvements to clinical care. In the context of UK-based primary care and similar health systems, it should not be implemented in the absence of care pathways[7] to ensure that case-found patients are promptly added to primary care COPD registers and receive appropriate management. Further trials investigating the effectiveness of COPD case finding are unlikely to be ethical in the absence of such pathways of care. Encouragingly, we identified one trial protocol that aims to evaluate COPD case finding in conjunction with an integrated care pathway in low and middle income countries[27] but more such trials will be needed to make firm recommendations.

In England, including patients on COPD primary care registers is associated with financial reimbursement through the Quality and Outcomes Framework.[28] This requires a number of care quality indicators, such as performing diagnostic spirometry and providing annual influenza vaccination, to be documented in electronic health records. This may at least partly explain why case-found patients who had been added to a disease register received significantly higher levels of COPD-related care than patients who had not been added. New indicators have recently been added to this scheme, including referral to a pulmonary rehabilitation programme for patients with MRC dyspnoea ≥3. This could potentially improve the levels of care provided to these patients.

Even in the presence of robust care pathways, further research and modelling will be needed to assess whether there is sufficient health service capacity to meet the demands of the additional cases of COPD that would be detected through targeted case finding. Most importantly, research is needed to empirically evaluate whether targeted case finding improves both short and long-term clinical outcomes and healthcare costs compared to usual care.



For peer review only - http://bmjopen.bmj.com/site/about/guidelines.xhtml

## Conclusions

Only a minority of patients with case-found COPD in primary care are likely to receive adequate levels of clinical care. Case finding is only likely to improve clinical care if patients with newly identified disease are promptly added to a primary care COPD register. This appears to be more likely to occur for patients who are current or former smokers, and have more severely impaired lung function. Further research is needed to model the impact of case finding on health service capacity, and to evaluate its effectiveness on clinical outcomes and costs.

For peer review only



For peer review only - http://bmjopen.bmj.com/site/about/guidelines.xhtml

## Funding statement

This paper presents independent research funded by the National Institute for Health Research (NIHR) under its Programme Grants for Applied Research Programme (Grant Reference Number RP-PG-0109-10061) and by the NIHR Birmingham Biomedical Research Centre at the University Hospitals Birmingham NHS Foundation Trust and the University of Birmingham. The views expressed are those of the author(s) and not necessarily those of the NIHR, the NHS, or the Department of Health and Social Care. TargetCOPD is part of The Birmingham Lung Improvement StudieS – BLISS.

## Competing interests

Peymane Adab, Rachel Jordan, and David Fitzmaurice were principle investigators for the Birmingham Lung Improvement StudieS (BLISS) programme, funded by the National Institute for Health Research (NIHR) under its Programme Grants for Applied Research (grant reference number RP-PG-0109-10061). We report grants from various NIHR studies. Peymane Adab is the Chair of the NIHR Public Health Research (PHR) Funding committee.

## Data sharing statement

All data requests should be submitted to authors RJ and PA for consideration.  Access to anonymised data may be granted following review.

## Contribution statement

RJ, PA, and DF were principal investigators on the TargetCOPD trial and were responsible for the follow-up of the trial. SH developed the protocol with input from RJ, PA and AD. SH conducted the analysis with input from RJ, PA and AS. AD and AE coordinated the collection of EHR follow-up data. KR developed the follow-up questionnaires with input from RJ, PA, AE, and SH. SH wrote the manuscript with input from all co-authors. All authors have contributed to and approved the final version.

## Patient and public involvement

The Birmingham Lung Improvement Studies (BLISS) Patient Advisory Group contributed to participant recruitment, design of questionnaires and interpretation of data.

For peer review only



For peer review only - http://bmjopen.bmj.com/site/about/guidelines.xhtml

# Figures

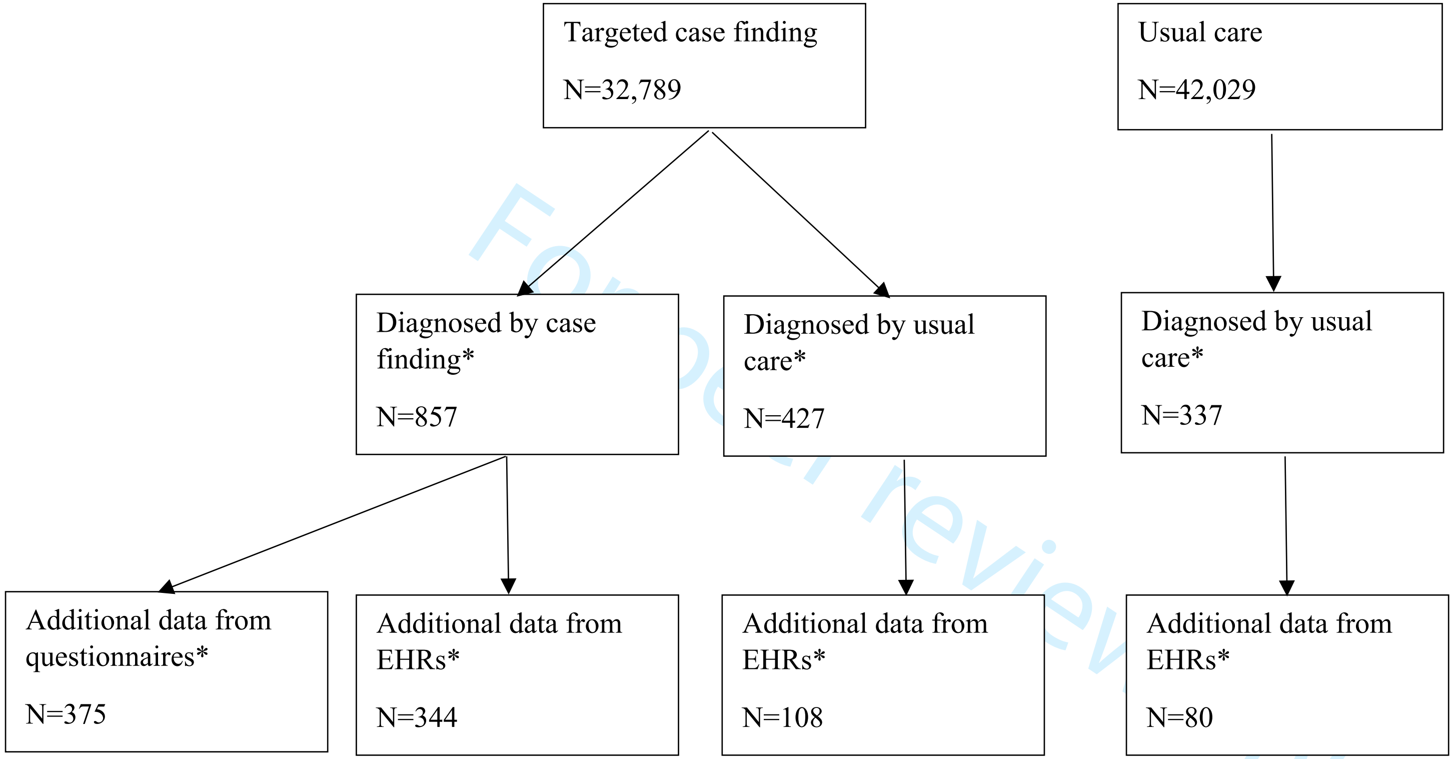


*Figure 1 Participant flow diagram (*included in the current study; EHR=electronic health record)*



For peer review only - http://bmjopen.bmj.com/site/about/guidelines.xhtml

# Tables

*Table 1 Characteristics* of participants newly diagnosed with COPD by case-finding and usual care*

| | | **Diagnostic route** | | | |
|---|---|---|---|---|---|
| | | **Case-finding (n=857)** | | **Usual care (n=764)** | |
| | | N | (%) | N | (%) |
| Age (years) | Mean (SD) | 63.8 | (9.6) | 63.7 | (9.7) |
| | 40-49 | 91 | (10.6) | 76 | (9.9) |
| | 50-59 | 190 | (22.2) | 184 | (24.1) |
| | 60-69 | 320 | (37.3) | 275 | (36.0) |
| | 70+ | 256 | (29.9) | 229 | (30.0) |
| Sex | Male | 489 | (57.1) | 418 | (54.7) |
| Ethnic group | White British | 556 | (64.9) | 507 | (66.4) |
| | Mixed | 5 | (0.6) | 5 | (0.7) |
| | Asian | 28 | (3.3) | 11 | (1.4) |
| | African/Caribbean | 14 | (1.6) | 24 | (3.1) |
| | Other | 17 | (2.0) | 3 | (0.4) |
| | Unknown | 237 | (27.7) | 214 | (28.0) |
| Smoking status | Current | 259 | (30.2) | 417 | (54.6) |
| | Former | 372 | (43.4) | 255 | (33.4) |
| | Never | 124 | (14.5) | 15 | (2.0) |
| | Unknown | 102 | (11.9) | 77 | (10.1) |
| Comorbidities** | Asthma | 71 | (20.6) | 46 | (24.5) |
| | IHD | 44 | (12.8) | 26 | (13.8) |
| | Heart failure | 9 | (2.6) | 7 | (3.7) |
| | Diabetes | 58 | (16.9) | 30 | (16.0) |
| | Depression | 19 | (5.5) | 19 | (10.1) |
| | Anxiety | 10 | (2.9) | 5 | (2.7) |
| | Osteoporosis | 12 | (3.5) | 8 | (4.3) |
| | Stroke | 6 | (1.7) | 6 | (3.2) |
| | No comorbidities | 172 | (50.0) | 84 | (44.7) |
| | 1 comorbidity | 123 | (35.8) | 71 | (37.8) |
| | ≥2 comorbidities | 49 | (5.7) | 33 | (4.3) |

*Based on data from electronic health records.
**Based on a subset of patients (344 patients diagnosed by case-finding and 188 diagnosed by usual care).



For peer review only - http://bmjopen.bmj.com/site/about/guidelines.xhtml

*Table 2 Multilevel logistic regression model* assessing the association between participant characteristics and likelihood of being listed on a COPD register among those with case-found COPD (n=754)*

| **Participant characteristics** | **aOR**** | **(95% CI)** | **p** |
|---|---|---|---|
| Age | 1.02 | (0.99, 1.05) | 0.139 |
| Sex (male) | 1.01 | (0.63, 1.60) | 0.976 |
| Smoking status | | | |
| Never smoked (reference) | | | |
| Ex-smoker | 6.32 | (1.88, 21.29) | 0.003 |
| Current smoker | 8.68 | (2.53, 29.82) | 0.001 |
| $FEV_1$ % predicted | 0.96 | (0.95, 0.98) | <0.001 |
| No. of self-reported comorbidities | 0.97 | (0.78, 1.20) | 0.766 |
| CAT score | 1.02 | (0.99, 1.05) | 0.218 |

*Using data from the main TargetCOPD trial dataset.
**aOR= adjusted odds ratio estimated by a multilevel logistic regression model, accounting for clustering by practice and adjusted for all variables listed in the table.
CAT=COPD assessment test, CI=confidence interval, $FEV_1$=forced expiratory volume in 1 second

*Table 3 Correlations between the percentages of case-found patients added to COPD registers and practice characteristics*

| **Practice characteristics** | **Correlation coefficient*** | **p** |
|---|---|---|
| % from non-white ethnic groups | 0.15 | 0.45 |
| Patient list size | 0.20 | 0.33 |
| Baseline prevalence of diagnosed COPD | 0.03 | 0.89 |
| Total number of case-found patients | -0.09 | 0.66 |
| Socioeconomic status | 0.33 | 0.09 |

*Pearson's correlation coefficient



For peer review only - http://bmjopen.bmj.com/site/about/guidelines.xhtml

*Table 4 Clinical care during the two year follow-up of participants with electronic health record data who were newly diagnosed with COPD by case-finding or usual care*

| | | Diagnostic route | | | |
|---|---|---|---|---|---|
| | | Case finding (n=344) | | Usual care (n=188) | |
| | | N* | (%) | N* | (%) |
| ***Clinical assessments*** | | | | | |
| MRC dyspnoea score recorded | | 98 | (28.5) | 171 | (91.0) |
| CAT score recorded | | 36 | (10.5) | 94 | (50.0) |
| Spirometry undertaken | | 48 | (14.0) | 79 | (42.0) |
| COPD severity recorded | | 33 | (9.6) | 96 | (51.1) |
| BMI recorded | | 244 | (70.9) | 168 | (89.4) |
| Oxygen saturations recorded | | 41 | (11.9) | 55 | (29.3) |
| Chest X-ray undertaken | | 13 | (3.8) | 9 | (4.8) |
| Depression screen undertaken | | 54 | (15.7) | 55 | (29.3) |
| ***Clinical interventions*** | | | | | |
| Listed on COPD register | | 78 | (22.7) | 175 | (93.1) |
| Care plan recorded | | 38 | (11.0) | 97 | (51.6) |
| Annual review undertaken | | 91 | (26.5) | 170 | (90.4) |
| Smoking cessation counselling | | 157 | (45.6) | 139 | (73.9) |
| Nicotine replacement therapy | | 27 | (7.8) | 17 | (9.0) |
| Influenza vaccination provided | | 240 | (69.8) | 138 | (73.4) |
| Pneumococcal vaccine provided | | 19 | (5.6) | 23 | (12.2) |
| Pulmonary rehabilitation provided | | 17 | (4.9) | 42 | (22.3) |
| Inhaler technique assessed | | 56 | (16.3) | 116 | (61.7) |
| Inhalers prescribed | Salbutamol | 128 | (37.2) | 152 | (80.9) |
| | Ipratropium | 5 | (1.5) | 10 | (5.3) |
| | Salmeterol | 3 | (0.9) | 10 | (5.3) |
| | Fluticasone | 3 | (0.9) | 1 | (0.5) |
| | Budesonide | 0 | (0.0) | 0 | (0.0) |
| | Beclometasone | 20 | (5.8) | 13 | (6.9) |
| | Fluticasone/salmeterol | 33 | (9.7) | 70 | (37.2) |
| | Budesonide/formoterol | 12 | (3.5) | 23 | (12.2) |
| | Any of the above inhalers | 134 | (39.0) | 163 | (86.7) |
| Antibiotic rescue pack | | 10 | (2.9) | 43 | (22.9) |
| Prednisolone | | 51 | (14.8) | 96 | (51.1) |
| **Clinical care score** | <5 | 225 | (65.4) | 17 | (9.0) |
| | 5 to 9 | 83 | (24.1) | 73 | (38.8) |
| | ≥10 | 33 | (9.6) | 98 | (52.1) |
| | Median (IQR) | 3 | (2 to 5) | 10 | (7 to 12) |

*Number of participants who received the clinical assessment or intervention.
BMI=body mass index, CAT=COPD Assessment Test, IQR=interquartile range, MRC=Medical Research Council

For peer review only

*Table 5 Clinical care of participants with follow-up questionnaire data who were newly diagnosed with COPD by case finding*

| | | Listed on COPD register | | | |
|---|---|---|---|---|---|
| | | Yes (n=78) | | No (n=297) | |
| | | N* | (%) | N* | (%) |
| Informed about COPD diagnosis | | 69 | (88.5) | 52 | (17.5) |
| Annual review undertaken | | 65 | (83.3) | 103 | (34.7) |
| Spirometry undertaken | | 68 | (87.2) | 111 | (37.4) |
| Inhaler technique assessed | | 37 | (47.4) | 72 | (24.2) |
| Antibiotics prescribed | | 14 | (17.9) | 18 | (6.1) |
| Steroids prescribed | | 13 | (16.7) | 14 | (4.7) |
| Influenza vaccine offered | | 77 | (98.7) | 242 | (81.5) |
| Influenza vaccine received | | 67 | (85.9) | 207 | (69.7) |
| Pneumococcal vaccine offered | | 44 | (56.4) | 128 | (43.1) |
| Pneumococcal vaccine received | | 43 | (55.1) | 121 | (40.7) |
| Pulmonary rehabilitation offered | | 4 | (5.1) | 5 | (1.7) |
| Attended pulmonary rehabilitation | | 3 | (3.8) | 6 | (2.0) |
| Smoking cessation advice given | | 44 | (56.4) | 103 | (34.7) |
| Smoking cessation support offered** | | 34 | (43.6) | 60 | (20.2) |
| Inhalers prescribed | SABA | 45 | (57.7) | 87 | (29.3) |
| | SAMA | 21 | (26.9) | 18 | (6.1) |
| | ICS | 5 | (6.4) | 23 | (7.7) |
| | LABA | 0 | (0.0) | 5 | (1.7) |
| | LAMA | 7 | (9.0) | 1 | (0.3) |
| | ICS/LABA | 17 | (21.8) | 40 | (13.5) |
| | LABA/LAMA | 2 | (2.6) | 2 | (0.7) |
| | Any of the above | 58 | (74.4) | 104 | (35.0) |
| Care plan provided | | 62 | (79.5) | 39 | (13.1) |
| **Clinical care score** | <5 | 9 | (11.5) | 214 | (72.1) |
| | 5 to 9 | 56 | (71.8) | 76 | (25.6) |
| | >=10 | 13 | (16.7) | 7 | (2.4) |
| | Median (IQR) | 8 | (6 to 9) | 3 | (2 to 5) |

*Number of participants self-reporting having received the clinical intervention.
**19/25 (76%) smokers listed on the COPD QOF register received smoking cessation support and 34/54 (63%) smokers not listed on the COPD QOF register had received this.
ICS=inhaled corticosteroid, IQR= interquartile range, LABA=long acting beta 2 agonist, LAMA= long acting muscarinic antagonist, SABA=short acting beta 2 antagonist, SAMA=short acting muscarinic antagonist

For peer review only

*Table 6 Multilevel linear regression model assessing the association between the clinical care score* and participant characteristics, among those with electronic health record data (n=467)*

| **Participant characteristics** | **aβ**** | **(95% CI)** | **p** |
|---|---|---|---|
| Age | 0.03 | (0.00, 0.05) | 0.050 |
| Sex (male) | -0.24 | (-0.71, 0.23) | 0.318 |
| Smoking status | | | |
| Ex-smoker | -0.13 | (-1.02, 0.77) | 0.781 |
| Current smoker | 0.79 | (-0.13, 1.71) | 0.094 |
| No. of comorbidities | 0.38 | (0.11, 0.65) | 0.007 |
| Case-found vs. routinely diagnosed | -0.69 | (-1.44, 0.07) | 0.076 |
| Listed on COPD register | 5.06 | (4.36, 5.75) | <0.001 |

*Based on the clinical care of participants 2 years after the close of the TargetCOPD trial
**Adjusted linear regression coefficient (this corresponds to the mean change in clinical care score for each unit rise in the independent variable), accounting for clustering by practice.

*Table 7 Linear regression model assessing the association between the COPD clinical care score and participant characteristics, among those with follow-up questionnaire data (n=293)*

| **Participant characteristics** | **aβ*** | **(95% CI)** | **p** |
|---|---|---|---|
| Age | 0.01 | (-0.03, 0.04) | 0.719 |
| Sex (male) | -0.49 | (-1.06, 0.09) | 0.096 |
| Smoking status | | | |
| Ex-smoker | 0.38 | (-0.49, 1.24) | 0.393 |
| Current smoker | 0.74 | (-0.27, 1.76) | 0.149 |
| FEV1 % predicted | -0.03 | (-0.04, -0.01) | <0.001 |
| No. of comorbidities | 0.38 | (0.10, 0.65) | 0.007 |
| CAT score | 0.05 | (0.01, 0.08) | 0.005 |
| Listed on COPD register | 3.48 | (2.81, 4.15) | <0.001 |

*Adjusted linear regression coefficient (this corresponds to the mean change in clinical care score for each unit rise in the independent variable).
CAT=COPD assessment test, CI=confidence interval, $FEV_1$=forced expiratory volume in 1 second



For peer review only - http://bmjopen.bmj.com/site/about/guidelines.xhtml

# References


1 GBD 2016 Causes of Death Collaborators M, Abajobir AA, Abbafati C, *et al.* Global, regional, and national age-sex specific mortality for 264 causes of death, 1980-2016: a systematic analysis for the Global Burden of Disease Study 2016. *Lancet (London, England)* 2017; **390**: 1151–210.

2 Soriano JB, Zielinski J, Price D. Screening for and early detection of chronic obstructive pulmonary disease. *Lancet (London, England)* 2009; **374**: 721–32.

3 Jordan RE, Lam KH, Cheng KK, *et al.* Case finding for chronic obstructive pulmonary disease: a model for optimising a targeted approach. *Thorax* 2010; **65**: 492–8.

4 Lambe T, Adab P, Jordan RE, *et al.* Model-based evaluation of the long-term cost-effectiveness of systematic case-finding for COPD in primary care. *Thorax* 2019; **74**: 730–9.

5 Criteria for appraising the viability, effectiveness and appropriateness of a screening programme. 2015. https://www.gov.uk/government/publications/evidence-review-criteria-national-screening-programmes/criteria-for-appraising-the-viability-effectiveness-and-appropriateness-of-a-screening-programme (accessed March 6, 2018).

6 Guirguis-Blake JM, Senger CA, Webber EM, Mularski RA, Whitlock EP. Screening for Chronic Obstructive Pulmonary Disease. *JAMA* 2016; **315**: 1378.

7 Yawn BP, Martinez FJ. POINT: Can Screening for COPD Improve Outcomes? Yes. Chest. 2020; **157**: 7–9.

8 Haroon SM, Jordan RE, O'beirne-Elliman J, Adab P. Effectiveness of case finding strategies for COPD in primary care: A systematic review and meta-analysis. *npj Prim Care Respir Med* 2015; **25**. DOI:10.1038/npjpcrm.2015.56.

9 Haroon S, Jordan R, Takwoingi Y, Adab P. Diagnostic accuracy of screening tests for COPD: A systematic review and meta-analysis. *BMJ Open* 2015; **5**. DOI:10.1136/bmjopen-2015-008133.

10 Jordan RERE, Adab P, Sitch A, *et al.* Targeted case finding for chronic obstructive pulmonary disease versus routine practice in primary care (TargetCOPD): a cluster-randomised controlled trial. *Lancet Respir Med* 2016; **4**: 720–30.

11 Miller MR, Hankinson J, Brusasco V, *et al.* Standardisation of spirometry. *Eur Respir J* 2005; **26**: 319–38.

12 Chronic obstructive pulmonary disease in over 16s: diagnosis and management | Guidance and guidelines | NICE. https://www.nice.org.uk/guidance/cg101/chapter/1-Guidance#managing-stable-copd (accessed Jan 19, 2018).

13 NHS Digital, 1 Trevelyan Square, Boar Lane, Leeds, LS1 6AE UK. Quality and Outcomes Framework. http://content.digital.nhs.uk/qof (accessed April 25, 2017).

14 Jordan RE, Adab P, Sitch A, *et al.* Targeted case finding for chronic obstructive pulmonary disease versus routine practice in primary care (TargetCOPD): a cluster-randomised controlled trial. *Lancet Respir Med* 2016; **4**. DOI:10.1016/S2213-2600(16)30149-7.

15 Ministry of Housing C and LG. English indices of deprivation 2015. https://www.gov.uk/government/statistics/english-indices-of-deprivation-2015 (accessed June 12, 2019).

16 Bertens LCM, Reitsma JB, van Mourik Y, *et al.* COPD detected with screening: impact on patient management and prognosis. *Eur Respir J* 2014; **44**: 1571–8.

17 Yawn BP, Duvall K, Peabody J, *et al.* The Impact of Screening Tools on Diagnosis of Chronic




For peer review only - http://bmjopen.bmj.com/site/about/guidelines.xhtml

Obstructive Pulmonary Disease in Primary Care. *Am J Prev Med* 2014; **47**: 563–75.

18 Wadland WC, Zubek VB, Clerisme-Beaty EM, Ríos-Bedoya CF, Yawn BP. Patient Factors Influencing Respiratory-Related Clinician Actions in Chronic Obstructive Pulmonary Disease Screening. *Am J Prev Med* 2017; **52**: 94–9.

19 Haroon S, Jordan RE, Fitzmaurice DA, Adab P. Case finding for COPD in primary care: A qualitative study of the views of health professionals. *Int J COPD* 2015; **10**. DOI:10.2147/COPD.S84247.

20 Enocson A, Jolly K, Jordan R, Fitzmaurice DA, Greenfield S, Adab P. Case-finding for COPD in primary care: a qualitative study of patients' perspectives. *Int J Chron Obstruct Pulmon Dis* 2018; **Volume 13**: 1623–32.

21 Sehl J, O'Doherty J, O'Connor R, O'Sullivan B, O'Regan A. Adherence to COPD management guidelines in general practice? A review of the literature. *Irish J Med Sci (1971 -)* 2018; **187**: 403–7.

22 Price D, West D, Brusselle G, *et al.* Management of COPD in the UK primary-care setting: an analysis of real-life prescribing patterns. *Int J Chron Obstruct Pulmon Dis* 2014; **9**: 889.

23 Chavez PC, Shokar NK. Diagnosis and Management of Chronic Obstructive Pulmonary Disease (COPD) in a Primary Care Clinic. *COPD J Chronic Obstr Pulm Dis* 2009; **6**: 446–51.

24 Primary care audit (Wales) 2015–17: Planning for every breath. London, 2017 https://www.rcplondon.ac.uk/projects/outputs/primary-care-audit-wales-2015-17-planning-every-breath (accessed Jan 19, 2018).

25 Mehuys E, Boussery K, Adriaens E, *et al.* COPD Management in Primary Care: An Observational, Community Pharmacy–Based Study. *Ann Pharmacother* 2010; **44**: 257–66.

26 Davis KJ, Landis SH, Oh Y-M, *et al.* Continuing to Confront COPD International Physician Survey: physician knowledge and application of COPD management guidelines in 12 countries. *Int J Chron Obstruct Pulmon Dis* 2015; **10**: 39–55.

27 Siddharthan T, Pollard SL, Quaderi SA, *et al.* Effectiveness-implementation of COPD case finding and self-management action plans in low- and middle-income countries: global excellence in COPD outcomes (GECo) study protocol. *Trials* 2018; **19**: 571.

28 Quality and Outcomes Framework Indicators. https://www.nice.org.uk/standards-and-indicators/qofindicators (accessed Jan 21, 2018).

For peer review only



For peer review only - http://bmjopen.bmj.com/site/about/guidelines.xhtml

*Supplementary Table 1 Clinical codes used to extract data on COPD-related care from GP electronic health records*

| **Variable** | **Variable (specific)** | **Read code v2** | **Read code v3** |
|---|---|---|---|
| Antibiotic rescue pack | | "8IEZ","8BMW" | "XaW9D","XaZp7" |
| Anxiety screening | | "6897","388N" | "Xab9E","XaIwe" |
| Body mass index | | "22K%" | "22K%" |
| COPD Care plan | | "8CMV","66YI" | "8CMV","XaIUt" |
| Chest x-ray | | "536" | "XE2az" |
| Clinical COPD Questionnaire (CCQ) | | "38Dd" | "XaR6n" |
| COPD Assessment Test (CAT) | | "38Dg" | "XaRFy" |
| COPD exception reporting | | "9h5%" | "XaJ4R","XaJ4l","XaJ4k" |
| COPD review | | "66YM" | "XaIet","XaXCb","XaXCa" |
| COPD severity | | "H39","H36","H37","H38" | "XaEIV","XaN4a","XaEIY","XaEIW" |
| Depression screening | | "6896%","6891%","388P","388w","E204","3885","38G5","388f" | "6891%","XaIwf","XaNkT","62T1","XaYS4","XaLDN" |
| Influenza vaccine | | "65E%","n47%","9OX5" | "65E%","n47%","XaIBI" |
| Inhaler technique assessed/status | | "6636","663I","663H","6637","66Y4" | "6636","663I","663H","6637","XaIQ0" |
| Prednisolone | | "fe6%" | "fe6%" |
| Beclometasone dipropionate | | "c61%","c66%" | "c61%","c66%" |
| Budesonide | | "c64%" | "c64%" |
| Budesonide + formoterol (Symbicort) | | "c67%" | "c67%" |
| Fluticasone + salmeterol (Seretide) | | "c1D%" | "c1D%" |
| Fluticasone propionate | | "c65%" | "c65%" |
| Indacaterol | | "c1b%" | "c1b%" |
| Ipratropium bromide | | "c31%" | "c31%" |
| Salbutamol | | "c13%","c1E%" | "c13%","c1E%" |
| Salmeterol | | "c19%" | "c19%" |
| Terbutaline | | "c144","C145","C146","C147","c148","c14d","c14f","c14g","c14i","c14j","c14k","c14t","c14u","c14v","c14w","c14x" | "c144","C145","C146","C147","c148","c14d","c14f","c14g","c14i","c14j","c14k","c14t","c14u","c14v","c14w","c14x" |
| Aminophylline | | "c41%" | "c41%" |
| Theophylline | | "c43%" | "c43%" |
| MRC dyspnoea score | | "173H","173I","173J","173K","173L" | "XaIUl","XaIUm","XaIUn","XaIUo","XaIUi" |
| Oxygen saturation | | "44YA" | "X7708" |
| Oxygen therapy | | "8771%" | "8771%","X71a%","XALL8" |
| Palliative care patients | | "9EB5","8CM1%" | "XaJv2","XE2XB","XaCDx" |

For peer review only

| Variable | Variable (specific) | Read code v2 | Read code v3 |
|---|---|---|---|
| Physical activity level (GPPAQ) | | "138b","138X","138a","138Y" | "XaPPE","XaPP8","XaPPD","XaPPB" |
| Pneumococcal vaccine | | "6572","n4b%","8I3Q" | "6572","n4b%","XaIyy" |
| Pulmonary rehabilitation | | "8H7u","8FA%","9NSL","8I86","8IA9" | "XaIf9","XaIUp","XaIUq","XS7qP","XaIUs" ,"XaZuM","XabGM","XaNQU" |
| Smoking cessation - medication | Bupropion / Nicotine replacement therapy / Varenicline | "du6%", "du3%","du7%", "du8%" | "du6%", "du3%","du7%", "du8%" |
| Smoking cessation | Declined smoking cessation support | "9NdZ" | "XaRFh" |
| Smoking cessation | Smoking cessation advice | "8CAL" | "Ua1Nz" |
| Smoking status | Current/ex/never | "137%" | "137%" |
| Spirometry | FEV1: actual / predicted / % predicted, FEV1/FVC ratio: actual / predicted / % predicted, FVC: actual / predicted / % predicted | "339S","339O","339P","339R","339M","339N","3396%","339Q" | "XaEFz","X77Qu","XaCJK","XaEFy","X77Ra","XaCFR","3396%","XaQmT","XaPpI","XaCJL","XaJ3K" |
| Spirometry | | "745D4","8HRC" | "XaXeg","XaK02" |

For peer review only

*Supplementary Table 2 Clinical codes used to define a clinician diagnosis of COPD*

| | |
|---|---|
| Clinical system: EMIS Web and LV | |
| H31 | Chronic bronchitis |
| H32 | Emphysema |
| H36 | Mild chronic obstructive pulmonary disease |
| H37 | Moderate chronic obstructive pulmonary disease |
| H38 | Severe chronic obstructive pulmonary disease |
| H39 | Very severe chronic obstructive pulmonary disease |
| H3A | End stage chronic obstructive airways disease |
| H3y | Other specific chronic obstructive airways disease |
| H3z | Chronic obstructive airways disease |
| Clinical system: System One | |
| H3 | COPD read codes in a cluster |

For peer review only

*Supplementary Table 3 Participant characteristics of those with and without additional electronic health record (EHR) or questionnaire data*

| **Participant characteristics** | **With additional EHR data** | **With follow-up questionnaire data** | **Without additional EHR or follow-up questionnaire data** |
|---|---|---|---|
| Mean age (SD) | 63.8 (9.4) | 64.7 (8.9) | 63.2 (10.2) |
| % Male | 62.2 | 58.7 | 54.2 |
| % Current smoker | 37.7 | 29.2 | 37.3 |
| Mean $FEV_1$ % predicted (SD) | 86.3 (18.1) | 87.8 (17.9) | 84.8 (18.4) |

*Supplementary Table 4 Participant characteristics of those with questionnaire data (n=375)*

| **Participant characteristics** | | **N** | **(%)** |
|---|---|---|---|
| Median age (IQR) | | 65.8 | (58.8-71.0) |
| Sex | Male | 220 | (58.7) |
| Smoking status | Current | 77 | (20.5) |
| | Former | 202 | (53.9) |
| | Never | 41 | (10.9) |
| $FEV_1$ % predicted | Mean | 87.8 | (17.9) |
| | ≥80 | 249 | (66.4) |
| | 50-79 | 121 | (32.3) |
| | <50 | 5 | (1.3) |
| Comorbidities | Asthma | 31 | (8.3) |
| | Ischaemic heart disease | 22 | (5.9) |
| | Diabetes | 20 | (5.3) |
| | Depression | 5 | (1.3) |
| | Anxiety | 6 | (1.6) |
| CAT score (median, IQR) | | 12 | (7-19) |
| Symptoms | MRC dyspnoea ≥3 | 148 | (39.5) |
| | Chronic cough | 128 | (34.1) |
| | Phlegm | 194 | (51.7) |
| | Wheeze | 274 | (73.1) |

CAT=COPD Assessment Test, FEV1=forced expiratory volume in 1 second

For peer review only

*Supplementary Table 5 Clinical care* of participants with electronic health record data who were newly diagnosed with COPD by case-finding and usual care*

| | | Diagnostic approach | | | |
|---|---|---|---|---|---|
| | | Case finding (n=341) | | Usual care (n=188) | |
| | | N** | (%) | N** | (%) |
| ***Clinical assessment*** | | | | | |
| MRC score recorded | | 107 | (31.4) | 176 | (93.6) |
| CAT score recorded | | 39 | (11.4) | 102 | (54.3) |
| Spirometry undertaken | | 101 | (29.6) | 147 | (78.2) |
| COPD severity recorded | | 48 | (14.1) | 118 | (62.8) |
| BMI recorded | | 277 | (81.2) | 178 | (94.7) |
| Oxygen saturations recorded | | 50 | (14.7) | 66 | (35.1) |
| CXR undertaken | | 22 | (6.5) | 20 | (10.6) |
| Depression screen undertaken | | 71 | (20.8) | 64 | (34.0) |
| ***Clinical intervention*** | | | | | |
| Listed on COPD register | | 78 | (22.9) | 175 | (93.1) |
| Care plan recorded | | 41 | (12.0) | 119 | (63.3) |
| Annual review undertaken | | 95 | (27.9) | 176 | (93.6) |
| Smoking cessation counselling provided | | 185 | (54.3) | 154 | (81.9) |
| Nicotine replacement therapy | | 39 | (11.4) | 29 | (15.4) |
| Influenza vaccination provided | | 245 | (71.8) | 144 | (76.6) |
| Pneumococcal vaccine provided | | 29 | (8.5) | 28 | (14.9) |
| Pulmonary rehabilitation provided | | 20 | (5.9) | 52 | (27.7) |
| Inhaler technique assessed | | 73 | (21.4) | 140 | (74.5) |
| Inhalers prescribed | Salbutamol | 146 | (42.8) | 167 | (88.8) |
| | Ipratropium | 8 | (2.3) | 15 | (8.0) |
| | Salmeterol | 3 | (0.9) | 15 | (8.0) |
| | Fluticasone | 3 | (0.9) | 1 | (0.5) |
| | Budesonide | 1 | (0.3) | 0 | (0.0) |
| | Beclometasone | 26 | (7.6) | 23 | (12.2) |
| | Fluticasone/salmeterol | 37 | (10.9) | 77 | (41.0) |
| | Budesonide/formoterol | 12 | (3.5) | 26 | (13.8) |
| | Any of the above inhalers | 151 | (44.3) | 172 | (91.5) |
| Antibiotic rescue pack | | 11 | (3.2) | 48 | (25.5) |
| Prednisolone | | 69 | (20.2) | 114 | (60.6) |
| **Clinical care score** | <5 | 225 | (66.0) | 17 | (9.0) |
| | 5 to 9 | 83 | (24.3) | 73 | (38.8) |
| | ≥10 | 33 | (9.7) | 98 | (52.1) |
| | Median (IQR) | 4 | (2 to 6) | 11 | (9 to 13) |

*Includes data from the start up to 2 years following the end of the TargetCOPD trial
**Number of participants who received the clinical assessment or intervention
BMI=body mass index, CAT=COPD Assessment Test, IQR=interquartile range, MRC=Medical Research Council

For peer review only

*Supplementary Table 6 Multilevel linear regression model assessing the association between the clinical care score* and participant characteristics, among those with electronic health record data (n=467)*

| **Participant characteristics** | **aβ*** | **(95% CI)** | **p** |
|---|---|---|---|
| Age | 0.03 | (0.00, 0.05) | 0.022 |
| Sex (male) | -0.35 | (-0.80, 0.09) | 0.121 |
| Smoking status | | | |
| Ex-smoker | 0.01 | (-0.84, 0.86) | 0.987 |
| Current smoker | 1.09 | (0.21, 1.97) | 0.015 |
| No. of comorbidities | 0.32 | (0.09, 0.55) | 0.006 |
| Case-found vs. routinely diagnosed | -0.48 | (-1.21, 0.24) | 0.189 |
| Listed on COPD register | 6.15 | (5.49, 6.82) | <0.001 |

* Includes data from the start up to 2 years following the end of the TargetCOPD trial

For peer review only

**STROBE 2007 (v4) checklist of items to be included in reports of observational studies in epidemiology***

**Checklist for cohort, case-control, and cross-sectional studies (combined)**

| Section/Topic | Item # | Recommendation | Reported on page # |
|---|---|---|---|
| **Title and abstract** | 1 | (*a*) Indicate the study's design with a commonly used term in the title or the abstract | 1 |
| | | (*b*) Provide in the abstract an informative and balanced summary of what was done and what was found | 2 |
| **Introduction** | | | |
| Background/rationale | 2 | Explain the scientific background and rationale for the investigation being reported | 5 |
| Objectives | 3 | State specific objectives, including any pre-specified hypotheses | 5, 6 |
| **Methods** | | | |
| Study design | 4 | Present key elements of study design early in the paper | 7 |
| Setting | 5 | Describe the setting, locations, and relevant dates, including periods of recruitment, exposure, follow-up, and data collection | 8 |
| Participants | 6 | (*a*) *Cohort study*—Give the eligibility criteria, and the sources and methods of selection of participants. Describe methods of follow-up<br>*Case-control study*—Give the eligibility criteria, and the sources and methods of case ascertainment and control selection. Give the rationale for the choice of cases and controls<br>*Cross-sectional study*—Give the eligibility criteria, and the sources and methods of selection of participants | 8 |
| | | (*b*) *Cohort study*—For matched studies, give matching criteria and number of exposed and unexposed<br>*Case-control study*—For matched studies, give matching criteria and the number of controls per case | N/A |
| Variables | 7 | Clearly define all outcomes, exposures, predictors, potential confounders, and effect modifiers. Give diagnostic criteria, if applicable | 8, 9 |
| Data sources/ measurement | 8* | For each variable of interest, give sources of data and details of methods of assessment (measurement). Describe comparability of assessment methods if there is more than one group | 8, 9 |
| Bias | 9 | Describe any efforts to address potential sources of bias | 9 |
| Study size | 10 | Explain how the study size was arrived at | 7, 8 |
| Quantitative variables | 11 | Explain how quantitative variables were handled in the analyses. If applicable, describe which groupings were chosen and why | 9 |
| Statistical methods | 12 | (*a*) Describe all statistical methods, including those used to control for confounding | 9 |
| | | (*b*) Describe any methods used to examine subgroups and interactions | N/A |
| | | (*c*) Explain how missing data were addressed | 9 |
| | | (*d*) *Cohort study*—If applicable, explain how loss to follow-up was addressed<br>*Case-control study*—If applicable, explain how matching of cases and controls was addressed | N/A |

For peer review only

| | | | |
|---|---|---|---|
| | | *Cross-sectional study*—If applicable, describe analytical methods taking account of sampling strategy | |
| | | (*e*) Describe any sensitivity analyses | 9 |
| **Results** | | | |
| Participants | 13* | (a) Report numbers of individuals at each stage of study—eg numbers potentially eligible, examined for eligibility, confirmed eligible, included in the study, completing follow-up, and analysed | 11 |
| | | (b) Give reasons for non-participation at each stage | N/A |
| | | (c) Consider use of a flow diagram | 20 |
| Descriptive data | 14* | (a) Give characteristics of study participants (eg demographic, clinical, social) and information on exposures and potential confounders | 11 |
| | | (b) Indicate number of participants with missing data for each variable of interest | 21 |
| | | (c) *Cohort study*—Summarise follow-up time (eg, average and total amount) | 7 |
| Outcome data | 15* | *Cohort study*—Report numbers of outcome events or summary measures over time | 11 |
| | | *Case-control study*—Report numbers in each exposure category, or summary measures of exposure | N/A |
| | | *Cross-sectional study*—Report numbers of outcome events or summary measures | N/A |
| Main results | 16 | (*a*) Give unadjusted estimates and, if applicable, confounder-adjusted estimates and their precision (eg, 95% confidence interval). Make clear which confounders were adjusted for and why they were included | 12, 13, 22, 25 |
| | | (*b*) Report category boundaries when continuous variables were categorized | N/A |
| | | (*c*) If relevant, consider translating estimates of relative risk into absolute risk for a meaningful time period | N/A |
| Other analyses | 17 | Report other analyses done—eg analyses of subgroups and interactions, and sensitivity analyses | 13 |
| **Discussion** | | | |
| Key results | 18 | Summarise key results with reference to study objectives | 14 |
| Limitations | 19 | Discuss limitations of the study, taking into account sources of potential bias or imprecision. Discuss both direction and magnitude of any potential bias | 16 |
| Interpretation | 20 | Give a cautious overall interpretation of results considering objectives, limitations, multiplicity of analyses, results from similar studies, and other relevant evidence | 17 |
| Generalisability | 21 | Discuss the generalisability (external validity) of the study results | 17 |
| **Other information** | | | |
| Funding | 22 | Give the source of funding and the role of the funders for the present study and, if applicable, for the original study on which the present article is based | 19 |

*Give information separately for cases and controls in case-control studies and, if applicable, for exposed and unexposed groups in cohort and cross-sectional studies.

**Note:** An Explanation and Elaboration article discusses each checklist item and gives methodological background and published examples of transparent reporting. The STROBE checklist is best used in conjunction with this article (freely available on the Web sites of PLoS Medicine at http://www.plosmedicine.org/, Annals of Internal Medicine at http://www.annals.org/, and Epidemiology at http://www.epidem.com/). Information on the STROBE Initiative is available at www.strobe-statement.org.

For peer review only

BMJ Open

# The impact of COPD case finding on clinical care: a prospective analysis of the TargetCOPD trial

| | |
|---|---|
| Journal: | *BMJ Open* |
| Manuscript ID | bmjopen-2020-038286.R1 |
| Article Type: | Original research |
| Date Submitted by the Author: | 22-Jun-2020 |
| Complete List of Authors: | Haroon, Shamil; University of Birmingham,<br>Adab, Peymane; University of Birmingham, Public Health<br>Dickens, Andrew; University of Birmingham, Institute of Applied Health Research<br>Sitch, Alice; University of Birmingham, Public Health<br>Rai, Kiran; University of Birmingham, Institute of Applied Health Research<br>Enocson, Alexandra; University of Birmingham, Applied Health Research<br>Fitzmaurice, David; University of Warwick Warwick Medical School<br>Jordan, Rachel; University of Birmingham, Public Health and Epidemiology |
| <b>Primary Subject Heading</b>: | Respiratory medicine |
| Secondary Subject Heading: | General practice / Family practice, Public health |
| Keywords: | Chronic airways disease < THORACIC MEDICINE, PRIMARY CARE, PUBLIC HEALTH |
| | |

SCHOLARONE™
Manuscripts

*I, the Submitting Author has the right to grant and does grant on behalf of all authors of the Work (as defined in the below author licence), an exclusive licence and/or a non-exclusive licence for contributions from authors who are: i) UK Crown employees; ii) where BMJ has agreed a CC-BY licence shall apply, and/or iii) in accordance with the terms applicable for US Federal Government officers or employees acting as part of their official duties; on a worldwide, perpetual, irrevocable, royalty-free basis to BMJ Publishing Group Ltd ("BMJ") its licensees and where the relevant Journal is co-owned by BMJ to the co-owners of the Journal, to publish the Work in this journal and any other BMJ products and to exploit all rights, as set out in our licence.*

*The Submitting Author accepts and understands that any supply made under these terms is made by BMJ to the Submitting Author unless you are acting as an employee on behalf of your employer or a postgraduate student of an affiliated institution which is paying any applicable article publishing charge ("APC") for Open Access articles. Where the Submitting Author wishes to make the Work available on an Open Access basis (and intends to pay the relevant APC), the terms of reuse of such Open Access shall be governed by a Creative Commons licence – details of these licences and which Creative Commons licence will apply to this Work are set out in our licence referred to above.*

*Other than as permitted in any relevant BMJ Author's Self Archiving Policies, I confirm this Work has not been accepted for publication elsewhere, is not being considered for publication elsewhere and does not duplicate material already published. I confirm all authors consent to publication of this Work and authorise the granting of this licence.*

For peer review only

# The impact of COPD case finding on clinical care: a prospective analysis of the TargetCOPD trial

Authors: Shamil Haroon* (PhD), Peymané Adab* (MD), Andrew P. Dickens* (PhD), Alice J. Sitch*† (PhD), Kiran Rai* (PhD), Alexandra Enocson* (PhD), David A. Fitzmaurice‡ (MD), and Rachel E. Jordan* (PhD)

*Institute of Applied Health Research, College of Medical and Dental Sciences, University of Birmingham, Edgbaston, Birmingham, B15 2TT, UK.

†NIHR Birmingham Biomedical Research Centre, University Hospitals Birmingham NHS Foundation Trust and University of Birmingham, UK.

‡Warwick Medical School, University of Warwick, Coventry, CV4 7AL, UK.

Corresponding authors: Rachel Jordan (r.e.jordan@bham.ac.uk), T: +44 121 414 6775

Shamil Haroon (s.haroon@bham.ac.uk), T: +44 121 414 5042

Word count: Abstract- 274

Main text- 3610

Key words: COPD, case finding, screening, primary care, clinical management

For peer review only



For peer review only - http://bmjopen.bmj.com/site/about/guidelines.xhtml

# Abstract

## Objectives

To investigate the impact of COPD case finding on clinical care.

## Design

We conducted a prospective observational analysis of data from TargetCOPD, a pragmatic cluster RCT in primary care in the West Midlands, UK. This compared alternative methods of COPD case finding against usual care. Data were extracted from electronic healthcare records and self-reported questionnaires for a subset of patients with newly diagnosed COPD.

## Setting

50 general practices that participated in the TargetCOPD trial.

## Participants

Patients aged 40 to 79 years newly identified with COPD by targeted case finding or by usual care, from 10th August 2012 to 22nd June 2014.

## Primary and secondary outcome measures

The primary outcome was addition to a COPD register by the end of the trial. The secondary outcome was a clinical care score, derived from the sum of clinical assessments and relevant interventions. Associations between participant characteristics and the primary and secondary outcomes were assessed using multilevel regression.

## Results

857 patients identified with COPD by case finding and 764 by usual care were included. Only 21.2% of case-found patients had been added to a COPD register, compared to 92.7% of those diagnosed by usual care. The odds of being added were greater in smokers (adjusted odds ratio 8.68, 95% CI 2.53-29.8), and in those with lower percentage of predicted $FEV_1$ (adjusted OR 0.96 per percentage rise,




For peer review only - http://bmjopen.bmj.com/site/about/guidelines.xhtml

95% CI 0.95-0.98). Patients who had been added to a COPD register had a significantly higher clinical care score (mean difference 5.06, 95% CI 4.36-5.75).

## Conclusions

Only one in five case-found patients had been registered with COPD. Patients added to a COPD register received significantly higher levels of appropriate clinical care.

For peer review only



For peer review only - http://bmjopen.bmj.com/site/about/guidelines.xhtml

# Strengths and limitations of this study

- We conducted a detailed evaluation of the respiratory care received by patients with case-found COPD and compared this to that received by patients diagnosed by usual care over the same period.
- The analyses adjusted for a number of important confounding factors, including measures of disease severity.
- The analysis was done on only a subset of trial participants.
- Electronic health record data quality was highly dependent on clinical coding practices.
- The clinical care score represents a relatively crude estimate of overall respiratory care and individual components of the score were not weighted for their relative importance.

For peer review only

# Introduction

COPD is the third leading cause of death worldwide and a major cause of disability.[1] There is a large burden of undiagnosed COPD globally and a number of experts and policy makers have called for early detection and screening.[2] This has the potential to prevent a significant burden of premature morbidity and mortality. A targeted case finding approach in England could reduce COPD hospitalisations by an estimated 3300 per year and prevent almost 3000 premature deaths over 3 years.[3] A recent model-based evaluation also concluded that systematic case finding for COPD could be cost-effective in the long term.[4] However, this is based on the assumption that case-found patients go on to receive improved clinical care.

The UK National Screening Committee (NSC) requires evidence from high quality randomised controlled trials (RCTs) that a screening programme reduces mortality or morbidity and that the benefits outweigh any harms, before recommending population-based screening.[5] A systematic review for the US Preventive Services Task Force concluded that screening asymptomatic people for COPD could not be recommended because of a lack of evidence that it improves health-related quality of life, morbidity or mortality.[6] However, this does not necessarily apply to patients with symptoms.[7]

A large number of studies have evaluated case finding approaches for COPD in primary care.[8,9] However, few have assessed the clinical outcomes of case-found patients compared to those diagnosed through usual care. TargetCOPD was a large cluster RCT in the West Midlands, UK which confirmed the effectiveness and cost-effectiveness of a targeted programme to identify new COPD cases in the primary care setting compared to usual care.[10]

Follow-up data were collected on participants identified with COPD during the trial period to assess the clinical interventions they subsequently received. The objective of this study was to describe the clinical care and management of patients newly identified with COPD by targeted case finding and compare this with those diagnosed by usual care over the same period. In addition, we assessed which



For peer review only - http://bmjopen.bmj.com/site/about/guidelines.xhtml

patient and practice-level factors were associated with better management of COPD among those who were newly diagnosed.



For peer review only - http://bmjopen.bmj.com/site/about/guidelines.xhtml

# Methods

## Study design

We undertook a prospective observational analysis of data on the clinical care of patients newly diagnosed with COPD in the TargetCOPD trial.[10] TargetCOPD was a cluster RCT comparing the effectiveness and cost-effectiveness of alternative approaches to targeted case finding against usual care on the identification of previously undiagnosed COPD.

Patients aged 40-79 years with no prior diagnosis of COPD were identified from electronic general practice registers. Those in the case finding arm were provided screening questionnaires by post and/or opportunistically when visiting their practice. Patients reporting respiratory symptoms were then invited for a spirometry assessment. Post-bronchodilator spirometry was performed by trained researchers according to American Thoracic Society and European Respiratory Society 2005 guidelines[11] using an ultrasonic flow head spirometer (Spiroson-AS, ndd Medical Technologies, Zurich, Switzerland).

The study team sent letters to GPs informing them of patients identified as likely to have COPD through case-finding (see below for definition) with advice to follow the relevant National Institute for Health and Care Excellence (NICE) guidelines.[12] Patients newly diagnosed either by case finding or by usual care during the trial period were tracked through GP records. The trial was active in each practice for a period of one year, with a staggered start from 10th August 2012 to 22nd June 2013.

From 6th October 2015 to 12th October 2016, data on the clinical care of COPD were extracted from the electronic healthcare records (EHRs) of a sample of eligible patients from both arms of the trial (clinical codes listed in Supplementary Table 1). All case-found patients who agreed to further study were sent questionnaires from 30th March to 11th April 2018 that included detailed questions about the management of their COPD.



For peer review only - http://bmjopen.bmj.com/site/about/guidelines.xhtml

## Setting

This analysis used data from 50 out of 54 general practices that participated in the TargetCOPD trial. Relevant data could not be extracted from four practices due to practice closures (n=3) and missing practice lists (n=1).

## Participants

In this study we included those aged 40 to 79 years at baseline who were newly identified with COPD by targeted case finding or by usual care, from 10$^{th}$ August 2012 to 22$^{nd}$ June 2014. Case-found COPD was defined as a post-bronchodilator forced expiratory volume in one second to forced vital capacity ratio ($FEV_1$/FVC) of less than 0.7 (in line with recommended UK guidelines at the time) in those who reported respiratory symptoms (chronic cough or phlegm for at least three months of the year for two or more years, wheeze in the previous 12 months or MRC grade 2 dyspnoea or higher). Newly diagnosed COPD by usual care was defined as a clinician diagnosis recorded on the EHR using pre-defined clinical codes (Supplementary Table 2) made independently of case finding. This included all patients diagnosed with COPD in the usual care arm and those diagnosed in the case finding arm prior to receiving a trial spirometry assessment.

## Outcomes

The primary outcome was the addition of patients to a COPD register by the close of the trial period. There is currently a contractual requirement for general practices in England to maintain a register of patients with COPD.[13] Addition to a register is clinician-led but supported by administrators.

The secondary outcome was a composite clinical care score, derived by summing the number of clinical assessments and interventions recorded (one point for each) from the end of the trial up to two years of follow-up. The components of the score were based on clinical assessments and interventions that were relevant to this patient group, based on NICE guidelines.[12] Clinical assessments included recording MRC dyspnoea score, COPD assessment test (CAT), post-bronchodilator spirometry, COPD severity, body mass index, oxygen saturation, chest X-ray, and screening for depression and anxiety. Clinical interventions included recording a care plan, annual review, smoking cessation



For peer review only - http://bmjopen.bmj.com/site/about/guidelines.xhtml

support (brief advice and nicotine replacement therapy [NRT] for current smokers), influenza and pneumococcal vaccination, referral for pulmonary rehabilitation, assessment of inhaler technique, and prescriptions of inhalers, antibiotics, and prednisolone. Sensitivity analyses included data from the beginning of the trial up to three years of follow-up.

## Other variables

Data on demographic characteristics, smoking status, and comorbidities (asthma, ischaemic heart disease, heart failure, diabetes, depression, anxiety, osteoporosis, and stroke) were also obtained for included participants from the main trial dataset. Practice level data were extracted including patient list size, socioeconomic status (based on the Index of Multiple Deprivation [IMD]), percentage of the registered patient list from non-white ethnicities, and the baseline percentage of patients already diagnosed with COPD (data provided by each practice).

## Statistical methods

The primary analysis used a multilevel logistic regression model to investigate the association between the odds of being added to a COPD register and patient characteristics, among those with case-found COPD. This included the practice as a cluster variable, and demographic and clinical characteristics as independent variables, adjusting for age, sex, smoking status, percentage of predicted $FEV_1$, number of self-reported comorbidities, and CAT score. A complete case analysis was performed due to low levels of missing data.

The secondary analysis used a multilevel linear regression model among participants diagnosed with COPD during the trial to assess associations between the clinical care score and patient characteristics, diagnostic route (case-found vs. usual care), and addition to a COPD register. The model was run separately using EHR and questionnaire data, with the latter restricted to participants with case-found COPD. Associations between the percentage of case-found patients added to COPD registers and practice characteristics were explored using Pearson correlation coefficients. All analyses were performed using Stata M/P version 14.2.



For peer review only - http://bmjopen.bmj.com/site/about/guidelines.xhtml

## Ethical approval

The Solihull Research Ethics Committee provided ethical approval for the TargetCOPD trial (IRAS, reference 11/WM/0403).

## Role of the funding source

The study sponsors had no role in the study design; in the collection, analysis, and interpretation of data; in the writing of the report; and in the decision to submit the paper for publication. The corresponding authors had full access to all the data in the study and have final responsibility for the decision to submit for publication.

For peer review only



For peer review only - http://bmjopen.bmj.com/site/about/guidelines.xhtml

# Results

## Participants

Over the trial period, 1621 people with previously undiagnosed COPD were identified in participating practices, including 857 through case finding, and 764 by usual care (337 of these from the usual care arm).[14] EHR data on clinical care were available for 532 patients who were identified with COPD through either case finding or usual care (figure 1). Among the 857 case-found patients, 375 (43.8%) returned questionnaires. The characteristics of participants with and without additional EHR and questionnaire data were broadly similar (Supplementary Table 3).

The mean age of participants newly diagnosed with COPD by case finding and usual care was similar (63.8 and 63.7 years, respectively), a similar proportion were male, and the majority (65.6%) were white British (Table 1). A greater proportion of participants diagnosed by usual care were current smokers compared with those diagnosed by case finding (54.6% vs. 30.2%), although the proportion with chronic conditions was similar. The sub-sample with case-found COPD who returned a questionnaire were slightly older, more likely to be male, and generally reported fewer comorbidities than the full sample of case-found patients (Supplementary Table 4).

## Practice characteristics

The mean practice list size was 5762 patients (SD 3482). The mean socioeconomic status (mean IMD score 36.7, SD 15.2) was similar to Birmingham as a whole (mean 37.8).[15] The majority of registered patients were white British (mean 80.5%, SD 20.5) and the mean prevalence of diagnosed COPD at the start of the trial was 1.6% (SD 0.6).

## Addition to COPD registers

Of the 857 patients with case-found COPD, only 182 (21.2%) had been added to a COPD register, compared to 708 out of 764 patients (92.7%) diagnosed by usual care. Among those with case-found COPD, the median time from the trial spirometry assessment to being added to a COPD register was 152 days (IQR 72 to 258).



For peer review only - http://bmjopen.bmj.com/site/about/guidelines.xhtml

Among case-found patients, the odds of being added to a COPD register were higher among current or former smokers (adjusted odds ratio 8.68, 95% CI 2.53 to 29.8, and 6.32, 95% CI 1.88 to 21.3, respectively), and those with a lower percentage of predicted $FEV_1$ (adjusted OR 0.96 per percentage rise, 95% CI 0.95 to 0.98; Table 2).

The median percentage of case-found patients added to a COPD register in each general practice was 14.5% (IQR 3.2% to 32.3%). Overall there were no significant correlations between the percentage of patients added to a COPD register and the measured practice characteristics (Table 3).

## Clinical care of newly diagnosed COPD

Among patients with EHR data, clinical assessments that were commonly performed within the two year follow-up period included measurement of BMI (77.4%) and documentation of the MRC dyspnoea score (50.6%; Table 4). Documentation of CAT score, disease severity, oxygen saturation, chest X-ray, and depression screening, was infrequent. All aspects of clinical assessment were more commonly performed for patients diagnosed through usual care than for those by case finding.

Therapies that were commonly delivered included influenza vaccination (69.8% vs. 73.4% for those diagnosed through case finding and usual care, respectively), smoking cessation counselling for current smokers (45.6% vs. 73.9%), and prescription of inhalers (39.0% vs. 86.7%). Prescription of NRT, pulmonary rehabilitation, and pneumococcal vaccination were infrequent. The median clinical care score was significantly higher among participants who had been diagnosed by usual care than those by case finding (10 vs. 3, respectively).

Among case-found patients with questionnaire data (n=375), those added to a COPD register (n=78 [20.8%]) were more likely to have been informed of their COPD diagnosis (88.5% vs 17.5%; Table 5). They were also more likely to have received a number of clinical interventions, including a care plan (79.5% vs 13.1%), influenza and pneumococcal vaccination (85.9% vs 69.7%, and 55.1% vs 40.7%, respectively), and prescriptions of inhalers (74.4% vs 35.0%). Very few had been offered or referred to a pulmonary rehabilitation service irrespective of whether they had been added to a COPD register.

For peer review only



For peer review only - http://bmjopen.bmj.com/site/about/guidelines.xhtml

## Factors associated with higher levels of clinical care

Using EHR data, patients who had been added to a COPD register had a clinical care score 5 points higher than those who had not (adjusted mean difference 5.06, 95% CI 4.36 to 5.75; Table 6). This was also found to a lesser extent for those with a higher number of comorbidities (adjusted mean difference 0.38 per additional comorbidity, 95% CI 0.11 to 0.65). These findings remained consistent in sensitivity analyses when data from the beginning of the trial were also included, although current smoking also became significantly associated with an increase in the clinical care score (1.09, 95% CI 0.21 to 1.97; Supplementary Tables 5 and 6).

Similarly, among those with questionnaire data, patients who had been added to a COPD register had a higher clinical care score than those who had not (adjusted mean difference 3.48, 95% CI 2.81 to 4.15). This was also true to a lesser extent for those who had a higher CAT score (0.05 per unit rise in CAT score, 95% CI 0.01 to 0.08), and lower percentage of predicted $FEV_1$ (-0.02 per percentage rise, 95% CI -0.03 to -0.01; Table 7).

For peer review only



For peer review only - http://bmjopen.bmj.com/site/about/guidelines.xhtml

# Discussion

## Main findings

Despite being symptomatic and eligible for clinical care, only one in five patients newly diagnosed with COPD through targeted case finding in primary care had been added to a COPD register by the close of the TargetCOPD trial, compared to more than 90% of those diagnosed by usual care. Addition to the register was more likely among current and former smokers, and those with poorer lung function. Practice characteristics did not correlate with the percentage of case-found patients added to COPD registers.

The clinical care of COPD was significantly more comprehensive for patients who had been diagnosed by usual care than those by case finding, receiving on average seven more clinical assessments or interventions. This was mainly because patients diagnosed by usual care were significantly more likely to be added to a COPD register. In addition, patients with a higher number of comorbidities, worse CAT scores, poorer lung function, and current smokers, were also more likely to receive a higher level of respiratory care.

Very few patients diagnosed by either approach had been offered or referred to pulmonary rehabilitation, irrespective of whether they had been added to a COPD register. Also, relatively few had been administered a pneumococcal vaccine or provided adequate smoking cessation support.

## Relationship to other studies

Only two published studies have evaluated the clinical care of patients newly diagnosed with COPD by case finding. Similar to our findings, these also showed that case-finding was not followed by adequate COPD management for most patients. One study based in the Netherlands examined community-dwelling frail patients aged 65 years and older with dyspnoea who had participated in a screening study for COPD and heart failure.[16] During six months of follow-up, only 13.7% (n=53) of the new cases of COPD had any changes made to respiratory drug prescriptions.



For peer review only - http://bmjopen.bmj.com/site/about/guidelines.xhtml

A large cluster RCT of COPD screening in the USA similarly found that respiratory-related clinical activity was limited following identification, with only 187 of 994 patients (19%) who screened positive for COPD receiving a respiratory intervention.[17] The study examined a limited number of clinical activities, which included referral for pulmonary function testing, referral to a respiratory specialist, and new respiratory medication prescriptions. The likelihood of receiving this care was associated with prior visits for respiratory issues and previous prescriptions of respiratory medications.[18]

A qualitative study exploring the views of healthcare providers within the TargetCOPD trial on screening, suggested that poor knowledge, lower perceived priority, and insufficient resources for COPD diagnosis and management were barriers to adequate COPD management in primary care.[19] A qualitative study with patients also found that GPs often lack the time to engage in case finding, that accessing primary care appointments can be difficult, and that communication about a diagnosis can often be lacking.[20] In addition, patients may occasionally be in denial of their respiratory symptoms or may not prioritise this over other health complaints. Case finding strategies will therefore need to address patient education on accessing health services more promptly for respiratory symptoms.

A recent literature review of COPD management in primary care found that there is significant variability in the provision of recommended treatments, with barriers including a lack of familiarity with clinical guidelines.[21] This is reflected in prescribing practices. An analysis of UK primary care prescribing data in 2014 among 24,957 patients found that COPD is often not managed according to GOLD or NICE guidelines.[22] 18% of GOLD stage 2 patients had received no treatment despite having symptoms, and a significant proportion had received inhaled corticosteroids irrespective of their disease severity and exacerbation history. Similar findings of inconsistent prescribing were found in a study assessing the management of COPD in a primary care clinic in the USA.[23]

More recently, the national COPD primary care audit in Wales found significant shortcomings in the clinical management of patients with COPD.[24] Only 12.5% of smokers had received smoking cessation support, 34.0% had not received an influenza vaccination and half of patients with MRC dyspnoea grades 3-5 had not been referred for pulmonary rehabilitation. A study of COPD care in



For peer review only - http://bmjopen.bmj.com/site/about/guidelines.xhtml

community pharmacies in Belgium similarly found low rates of influenza vaccination in patients younger than 65 years, poor medication adherence, and poor inhaler technique, among a significant proportion of COPD patients.[25] Findings from the Continuing to Confront COPD Survey suggested that there are likely to be significant shortcomings in the provision of guideline-recommended treatments among both primary and secondary care clinicians internationally.[26]

The UK National Screening Committee recommend that clinical service provision and patient outcomes should be optimised in all healthcare providers prior to participation in a screening programme.[5] These widespread gaps in care provision will need to be addressed before recommendations for targeted case finding can be made.

## Limitations

Limitations include the unavailability of data on all trial participants. Patient questionnaires were only available for 375 case-found patients and EHR data on clinical care for a subset of participants (344 out of 857 [40.1%] diagnosed by case finding and 188 out of 764 [24.6%] by usual care). However, the demographic and clinical characteristics of patients with and without relevant data were broadly similar, suggesting that the findings are likely to apply to the full study population (Supplementary Table 3). There was also limited data on disease severity, particularly in the usual care arm of the TargetCOPD trial. However, our analyses adjusted for measures of disease severity such as percentage of predicted $FEV_1$, CAT score, and number of comorbidities.

EHR data quality was highly dependent on clinical coding by participating practices. These were routinely collected health service data and were not specifically recorded for research purposes. Inadequate recording could lead to an underestimation of the level of clinical care provided. Furthermore, a significant amount of smoking cessation support in England is provided in community pharmacies, which would not necessarily be captured in GP records. However, self-reported questionnaire data on smoking cessation support were also collected from a subset of patients, and had broadly similar findings.



For peer review only - http://bmjopen.bmj.com/site/about/guidelines.xhtml

The clinical care score represents a relatively crude estimate of overall respiratory care and individual components of the score were not weighted for their relative importance. Some of the components reflect the management of COPD exacerbations, which may have differed in their incidence between case-found and routinely diagnosed patients. However, there are currently no validated methods or scores for quantifying overall levels of care for COPD and we therefore chose what we considered a reasonable and pragmatic approach. Finally, the primary outcome of addition to a COPD register is specific to the UK-context. However, our findings do suggest that COPD registries play an important role in supporting COPD management and could be encouraged elsewhere.

## Implications for practice, policy and research

Our findings suggest that COPD case finding in primary care is unlikely to result in improvements to clinical care. In the context of UK-based primary care and similar health systems, it should not be implemented in the absence of care pathways[7] to ensure that case-found patients are promptly added to primary care COPD registers and receive appropriate management. Further trials investigating the effectiveness of COPD case finding are unlikely to be ethical in the absence of such pathways of care. Encouragingly, we identified one trial protocol that aims to evaluate COPD case finding in conjunction with an integrated care pathway in low and middle income countries[27] but more such trials will be needed to make firm recommendations.

In England, including patients on COPD primary care registers is associated with financial reimbursement through the Quality and Outcomes Framework.[28] This requires a number of care quality indicators, such as performing diagnostic spirometry and providing annual influenza vaccination, to be documented in electronic health records. This may at least partly explain why case-found patients who had been added to a disease register received significantly higher levels of COPD-related care than patients who had not been added. New indicators have recently been added to this scheme, including referral to a pulmonary rehabilitation programme for patients with MRC dyspnoea ≥3. This could potentially improve the levels of care provided to these patients.



For peer review only - http://bmjopen.bmj.com/site/about/guidelines.xhtml

Even in the presence of robust care pathways, further research and modelling will be needed to assess whether there is sufficient health service capacity to meet the demands of the additional cases of COPD that would be detected through targeted case finding. Most importantly, research is needed to empirically evaluate whether targeted case finding improves both short and long-term clinical outcomes and healthcare costs compared to usual care.

## Conclusions

Only a minority of patients with case-found COPD in primary care are likely to receive adequate levels of clinical care. Case finding is only likely to improve clinical care if patients with newly identified disease are promptly added to a primary care COPD register. This appears to be more likely to occur for patients who are current or former smokers, and have more severely impaired lung function. Further research is needed to model the impact of case finding on health service capacity, and to evaluate its effectiveness on clinical outcomes and costs.

For peer review only

## Funding statement

This paper presents independent research funded by the National Institute for Health Research (NIHR) under its Programme Grants for Applied Research Programme (Grant Reference Number RP-PG-0109-10061) and by the NIHR Birmingham Biomedical Research Centre at the University Hospitals Birmingham NHS Foundation Trust and the University of Birmingham. The views expressed are those of the author(s) and not necessarily those of the NIHR, the NHS, or the Department of Health and Social Care. TargetCOPD is part of The Birmingham Lung Improvement StudieS – BLISS.

## Competing interests

Peymane Adab, Rachel Jordan, and David Fitzmaurice were principle investigators for the Birmingham Lung Improvement StudieS (BLISS) programme, funded by the National Institute for Health Research (NIHR) under its Programme Grants for Applied Research (grant reference number RP-PG-0109-10061). We report grants from various NIHR studies. Peymane Adab is the Chair of the NIHR Public Health Research (PHR) Funding committee.

## Data sharing statement

All data requests should be submitted to authors RJ and PA for consideration.  Access to anonymised data may be granted following review.

## Contribution statement

RJ, PA, and DF were principal investigators on the TargetCOPD trial and were responsible for the follow-up of the trial. SH developed the protocol with input from RJ, PA and AD. SH conducted the analysis with input from RJ, PA and AS. AD and AE coordinated the collection of EHR follow-up data. KR developed the follow-up questionnaires with input from RJ, PA, AE, and SH. SH wrote the manuscript with input from all co-authors. All authors have contributed to and approved the final version.

## Patient and public involvement

The Birmingham Lung Improvement Studies (BLISS) Patient Advisory Group contributed to participant recruitment, design of questionnaires and interpretation of data.

For peer review only



For peer review only - http://bmjopen.bmj.com/site/about/guidelines.xhtml

# Figures

Figure 1 Participant flow diagram (*included in the current study; EHR=electronic health record)

# Tables

*Table 1 Characteristics* of participants newly diagnosed with COPD by case-finding and usual care*

| | | Diagnostic route | | | |
|---|---|---|---|---|---|
| | | **Case-finding (n=857)** | | **Usual care (n=764)** | |
| | | N | (%) | N | (%) |
| Age (years) | Mean (SD) | 63.8 | (9.6) | 63.7 | (9.7) |
| | 40-49 | 91 | (10.6) | 76 | (9.9) |
| | 50-59 | 190 | (22.2) | 184 | (24.1) |
| | 60-69 | 320 | (37.3) | 275 | (36.0) |
| | 70+ | 256 | (29.9) | 229 | (30.0) |
| Sex | Male | 489 | (57.1) | 418 | (54.7) |
| Ethnic group | White British | 556 | (64.9) | 507 | (66.4) |
| | Mixed | 5 | (0.6) | 5 | (0.7) |
| | Asian | 28 | (3.3) | 11 | (1.4) |
| | African/Caribbean | 14 | (1.6) | 24 | (3.1) |
| | Other | 17 | (2.0) | 3 | (0.4) |
| | Unknown | 237 | (27.7) | 214 | (28.0) |
| Smoking status | Current | 259 | (30.2) | 417 | (54.6) |
| | Former | 372 | (43.4) | 255 | (33.4) |
| | Never | 124 | (14.5) | 15 | (2.0) |
| | Unknown | 102 | (11.9) | 77 | (10.1) |
| Comorbidities** | Asthma | 71 | (20.6) | 46 | (24.5) |
| | IHD | 44 | (12.8) | 26 | (13.8) |
| | Heart failure | 9 | (2.6) | 7 | (3.7) |
| | Diabetes | 58 | (16.9) | 30 | (16.0) |
| | Depression | 19 | (5.5) | 19 | (10.1) |
| | Anxiety | 10 | (2.9) | 5 | (2.7) |
| | Osteoporosis | 12 | (3.5) | 8 | (4.3) |
| | Stroke | 6 | (1.7) | 6 | (3.2) |
| | No comorbidities | 172 | (50.0) | 84 | (44.7) |
| | 1 comorbidity | 123 | (35.8) | 71 | (37.8) |
| | ≥2 comorbidities | 49 | (5.7) | 33 | (4.3) |

*Based on data from electronic health records.
**Based on a subset of patients (344 patients diagnosed by case-finding and 188 diagnosed by usual care).



For peer review only - http://bmjopen.bmj.com/site/about/guidelines.xhtml

*Table 2 Multilevel logistic regression model* assessing the association between participant characteristics and likelihood of being listed on a COPD register among those with case-found COPD (n=754)*

| Participant characteristics | aOR** | (95% CI) | p |
|---|---|---|---|
| Age | 1.02 | (0.99, 1.05) | 0.139 |
| Sex (male) | 1.01 | (0.63, 1.60) | 0.976 |
| Smoking status | | | |
| Never smoked (reference) | | | |
| Ex-smoker | 6.32 | (1.88, 21.29) | 0.003 |
| Current smoker | 8.68 | (2.53, 29.82) | 0.001 |
| $FEV_1$ % predicted | 0.96 | (0.95, 0.98) | <0.001 |
| No. of self-reported comorbidities | 0.97 | (0.78, 1.20) | 0.766 |
| CAT score | 1.02 | (0.99, 1.05) | 0.218 |

*Using data from the main TargetCOPD trial dataset.
**aOR= adjusted odds ratio estimated by a multilevel logistic regression model, accounting for clustering by practice and adjusted for all variables listed in the table.
CAT=COPD assessment test, CI=confidence interval, $FEV_1$=forced expiratory volume in 1 second

*Table 3 Correlations between the percentages of case-found patients added to COPD registers and practice characteristics*

| Practice characteristics | Correlation coefficient* | p |
|---|---|---|
| % from non-white ethnic groups | 0.15 | 0.45 |
| Patient list size | 0.20 | 0.33 |
| Baseline prevalence of diagnosed COPD | 0.03 | 0.89 |
| Total number of case-found patients | -0.09 | 0.66 |
| Socioeconomic status | 0.33 | 0.09 |

*Pearson's correlation coefficient



For peer review only - http://bmjopen.bmj.com/site/about/guidelines.xhtml

*Table 4 Clinical care during the two year follow-up of participants with electronic health record data who were newly diagnosed with COPD by case-finding or usual care*

| | | Diagnostic route | | | |
|---|---|---|---|---|---|
| | | Case finding (n=344) | | Usual care (n=188) | |
| | | N* | (%) | N* | (%) |
| ***Clinical assessments*** | | | | | |
| MRC dyspnoea score recorded | | 98 | (28.5) | 171 | (91.0) |
| CAT score recorded | | 36 | (10.5) | 94 | (50.0) |
| Spirometry undertaken | | 48 | (14.0) | 79 | (42.0) |
| COPD severity recorded | | 33 | (9.6) | 96 | (51.1) |
| BMI recorded | | 244 | (70.9) | 168 | (89.4) |
| Oxygen saturations recorded | | 41 | (11.9) | 55 | (29.3) |
| Chest X-ray undertaken | | 13 | (3.8) | 9 | (4.8) |
| Depression screen undertaken | | 54 | (15.7) | 55 | (29.3) |
| ***Clinical interventions*** | | | | | |
| Listed on COPD register | | 78 | (22.7) | 175 | (93.1) |
| Care plan recorded | | 38 | (11.0) | 97 | (51.6) |
| Annual review undertaken | | 91 | (26.5) | 170 | (90.4) |
| Smoking cessation counselling | | 157 | (45.6) | 139 | (73.9) |
| Nicotine replacement therapy | | 27 | (7.8) | 17 | (9.0) |
| Influenza vaccination provided | | 240 | (69.8) | 138 | (73.4) |
| Pneumococcal vaccine provided | | 19 | (5.6) | 23 | (12.2) |
| Pulmonary rehabilitation provided | | 17 | (4.9) | 42 | (22.3) |
| Inhaler technique assessed | | 56 | (16.3) | 116 | (61.7) |
| Inhalers prescribed | Salbutamol | 128 | (37.2) | 152 | (80.9) |
| | Ipratropium | 5 | (1.5) | 10 | (5.3) |
| | Salmeterol | 3 | (0.9) | 10 | (5.3) |
| | Fluticasone | 3 | (0.9) | 1 | (0.5) |
| | Budesonide | 0 | (0.0) | 0 | (0.0) |
| | Beclometasone | 20 | (5.8) | 13 | (6.9) |
| | Fluticasone/salmeterol | 33 | (9.7) | 70 | (37.2) |
| | Budesonide/formoterol | 12 | (3.5) | 23 | (12.2) |
| | Any of the above inhalers | 134 | (39.0) | 163 | (86.7) |
| Antibiotic rescue pack | | 10 | (2.9) | 43 | (22.9) |
| Prednisolone | | 51 | (14.8) | 96 | (51.1) |
| **Clinical care score** | <5 | 225 | (65.4) | 17 | (9.0) |
| | 5 to 9 | 83 | (24.1) | 73 | (38.8) |
| | ≥10 | 33 | (9.6) | 98 | (52.1) |
| | Median (IQR) | 3 | (2 to 5) | 10 | (7 to 12) |

*Number of participants who received the clinical assessment or intervention.
BMI=body mass index, CAT=COPD Assessment Test, IQR=interquartile range, MRC=Medical Research Council



For peer review only - http://bmjopen.bmj.com/site/about/guidelines.xhtml

*Table 5 Clinical care of participants with follow-up questionnaire data who were newly diagnosed with COPD by case finding*

| | | Listed on COPD register | | | |
|---|---|---|---|---|---|
| | | Yes (n=78) | | No (n=297) | |
| | | N* | (%) | N* | (%) |
| Informed about COPD diagnosis | | 69 | (88.5) | 52 | (17.5) |
| Annual review undertaken | | 65 | (83.3) | 103 | (34.7) |
| Spirometry undertaken | | 68 | (87.2) | 111 | (37.4) |
| Inhaler technique assessed | | 37 | (47.4) | 72 | (24.2) |
| Antibiotics prescribed | | 14 | (17.9) | 18 | (6.1) |
| Steroids prescribed | | 13 | (16.7) | 14 | (4.7) |
| Influenza vaccine offered | | 77 | (98.7) | 242 | (81.5) |
| Influenza vaccine received | | 67 | (85.9) | 207 | (69.7) |
| Pneumococcal vaccine offered | | 44 | (56.4) | 128 | (43.1) |
| Pneumococcal vaccine received | | 43 | (55.1) | 121 | (40.7) |
| Pulmonary rehabilitation offered | | 4 | (5.1) | 5 | (1.7) |
| Attended pulmonary rehabilitation | | 3 | (3.8) | 6 | (2.0) |
| Smoking cessation advice given | | 44 | (56.4) | 103 | (34.7) |
| Smoking cessation support offered** | | 34 | (43.6) | 60 | (20.2) |
| Inhalers prescribed | SABA | 45 | (57.7) | 87 | (29.3) |
| | SAMA | 21 | (26.9) | 18 | (6.1) |
| | ICS | 5 | (6.4) | 23 | (7.7) |
| | LABA | 0 | (0.0) | 5 | (1.7) |
| | LAMA | 7 | (9.0) | 1 | (0.3) |
| | ICS/LABA | 17 | (21.8) | 40 | (13.5) |
| | LABA/LAMA | 2 | (2.6) | 2 | (0.7) |
| | Any of the above | 58 | (74.4) | 104 | (35.0) |
| Care plan provided | | 62 | (79.5) | 39 | (13.1) |
| **Clinical care score** | <5 | 9 | (11.5) | 214 | (72.1) |
| | 5 to 9 | 56 | (71.8) | 76 | (25.6) |
| | >=10 | 13 | (16.7) | 7 | (2.4) |
| | Median (IQR) | 8 | (6 to 9) | 3 | (2 to 5) |

*Number of participants self-reporting having received the clinical intervention.
**19/25 (76%) smokers listed on the COPD QOF register received smoking cessation support and 34/54 (63%) smokers not listed on the COPD QOF register had received this.
ICS=inhaled corticosteroid, IQR= interquartile range, LABA=long acting beta 2 agonist, LAMA= long acting muscarinic antagonist, SABA=short acting beta 2 antagonist, SAMA=short acting muscarinic antagonist



For peer review only - http://bmjopen.bmj.com/site/about/guidelines.xhtml

*Table 6 Multilevel linear regression model assessing the association between the clinical care score* and participant characteristics, among those with electronic health record data (n=467)*

| Participant characteristics | aβ** | (95% CI) | p |
|---|---|---|---|
| Age | 0.03 | (0.00, 0.05) | 0.050 |
| Sex (male) | -0.24 | (-0.71, 0.23) | 0.318 |
| Smoking status | | | |
| Ex-smoker | -0.13 | (-1.02, 0.77) | 0.781 |
| Current smoker | 0.79 | (-0.13, 1.71) | 0.094 |
| No. of comorbidities | 0.38 | (0.11, 0.65) | 0.007 |
| Case-found vs. routinely diagnosed | -0.69 | (-1.44, 0.07) | 0.076 |
| Listed on COPD register | 5.06 | (4.36, 5.75) | <0.001 |

*Based on the clinical care of participants 2 years after the close of the TargetCOPD trial
**Adjusted linear regression coefficient (this corresponds to the mean change in clinical care score for each unit rise in the independent variable), accounting for clustering by practice.

*Table 7 Linear regression model assessing the association between the COPD clinical care score and participant characteristics, among those with case-found COPD and follow-up questionnaire data (n=293)*

| Participant characteristics | aβ* | (95% CI) | p |
|---|---|---|---|
| Age | 0.01 | (-0.03, 0.04) | 0.719 |
| Sex (male) | -0.49 | (-1.06, 0.09) | 0.096 |
| Smoking status | | | |
| Ex-smoker | 0.38 | (-0.49, 1.24) | 0.393 |
| Current smoker | 0.74 | (-0.27, 1.76) | 0.149 |
| FEV1 % predicted | -0.03 | (-0.04, -0.01) | <0.001 |
| No. of comorbidities | 0.38 | (0.10, 0.65) | 0.007 |
| CAT score | 0.05 | (0.01, 0.08) | 0.005 |
| Listed on COPD register | 3.48 | (2.81, 4.15) | <0.001 |

*Adjusted linear regression coefficient (this corresponds to the mean change in clinical care score for each unit rise in the independent variable).
CAT=COPD assessment test, CI=confidence interval, $FEV_1$=forced expiratory volume in 1 second

For peer review only



For peer review only - http://bmjopen.bmj.com/site/about/guidelines.xhtml

# References

1 GBD 2016 Causes of Death Collaborators M, Abajobir AA, Abbafati C, *et al.* Global, regional, and national age-sex specific mortality for 264 causes of death, 1980-2016: a systematic analysis for the Global Burden of Disease Study 2016. *Lancet (London, England)* 2017; **390**: 1151–210.

2 Soriano JB, Zielinski J, Price D. Screening for and early detection of chronic obstructive pulmonary disease. *Lancet (London, England)* 2009; **374**: 721–32.

3 Jordan RE, Lam KH, Cheng KK, *et al.* Case finding for chronic obstructive pulmonary disease: a model for optimising a targeted approach. *Thorax* 2010; **65**: 492–8.

4 Lambe T, Adab P, Jordan RE, *et al.* Model-based evaluation of the long-term cost-effectiveness of systematic case-finding for COPD in primary care. *Thorax* 2019; **74**: 730–9.

5 Criteria for appraising the viability, effectiveness and appropriateness of a screening programme. 2015. https://www.gov.uk/government/publications/evidence-review-criteria-national-screening-programmes/criteria-for-appraising-the-viability-effectiveness-and-appropriateness-of-a-screening-programme (accessed March 6, 2018).

6 Guirguis-Blake JM, Senger CA, Webber EM, Mularski RA, Whitlock EP. Screening for Chronic Obstructive Pulmonary Disease. *JAMA* 2016; **315**: 1378.

7 Yawn BP, Martinez FJ. POINT: Can Screening for COPD Improve Outcomes? Yes. Chest. 2020; **157**: 7–9.

8 Haroon SM, Jordan RE, O'beirne-Elliman J, Adab P. Effectiveness of case finding strategies for COPD in primary care: A systematic review and meta-analysis. *npj Prim Care Respir Med* 2015; **25**. DOI:10.1038/npjpcrm.2015.56.

9 Haroon S, Jordan R, Takwoingi Y, Adab P. Diagnostic accuracy of screening tests for COPD: A systematic review and meta-analysis. *BMJ Open* 2015; **5**. DOI:10.1136/bmjopen-2015-008133.

10 Jordan RERE, Adab P, Sitch A, *et al.* Targeted case finding for chronic obstructive pulmonary disease versus routine practice in primary care (TargetCOPD): a cluster-randomised controlled trial. *Lancet Respir Med* 2016; **4**: 720–30.

11 Miller MR, Hankinson J, Brusasco V, *et al.* Standardisation of spirometry. *Eur Respir J* 2005; **26**: 319–38.

12 Chronic obstructive pulmonary disease in over 16s: diagnosis and management | Guidance and guidelines | NICE. https://www.nice.org.uk/guidance/cg101/chapter/1-Guidance#managing-stable-copd (accessed Jan 19, 2018).

13 NHS Digital, 1 Trevelyan Square, Boar Lane, Leeds, LS1 6AE UK. Quality and Outcomes Framework. http://content.digital.nhs.uk/qof (accessed April 25, 2017).

14 Jordan RE, Adab P, Sitch A, *et al.* Targeted case finding for chronic obstructive pulmonary disease versus routine practice in primary care (TargetCOPD): a cluster-randomised controlled trial. *Lancet Respir Med* 2016; **4**. DOI:10.1016/S2213-2600(16)30149-7.

15 Ministry of Housing C and LG. English indices of deprivation 2015. https://www.gov.uk/government/statistics/english-indices-of-deprivation-2015 (accessed June 12, 2019).

16 Bertens LCM, Reitsma JB, van Mourik Y, *et al.* COPD detected with screening: impact on patient management and prognosis. *Eur Respir J* 2014; **44**: 1571–8.

17 Yawn BP, Duvall K, Peabody J, *et al.* The Impact of Screening Tools on Diagnosis of Chronic



For peer review only - http://bmjopen.bmj.com/site/about/guidelines.xhtml

Obstructive Pulmonary Disease in Primary Care. *Am J Prev Med* 2014; **47**: 563–75.

18 Wadland WC, Zubek VB, Clerisme-Beaty EM, Ríos-Bedoya CF, Yawn BP. Patient Factors Influencing Respiratory-Related Clinician Actions in Chronic Obstructive Pulmonary Disease Screening. *Am J Prev Med* 2017; **52**: 94–9.

19 Haroon S, Jordan RE, Fitzmaurice DA, Adab P. Case finding for COPD in primary care: A qualitative study of the views of health professionals. *Int J COPD* 2015; **10**. DOI:10.2147/COPD.S84247.

20 Enocson A, Jolly K, Jordan R, Fitzmaurice DA, Greenfield S, Adab P. Case-finding for COPD in primary care: a qualitative study of patients' perspectives. *Int J Chron Obstruct Pulmon Dis* 2018; **Volume 13**: 1623–32.

21 Sehl J, O'Doherty J, O'Connor R, O'Sullivan B, O'Regan A. Adherence to COPD management guidelines in general practice? A review of the literature. *Irish J Med Sci (1971 -)* 2018; **187**: 403–7.

22 Price D, West D, Brusselle G, *et al.* Management of COPD in the UK primary-care setting: an analysis of real-life prescribing patterns. *Int J Chron Obstruct Pulmon Dis* 2014; **9**: 889.

23 Chavez PC, Shokar NK. Diagnosis and Management of Chronic Obstructive Pulmonary Disease (COPD) in a Primary Care Clinic. *COPD J Chronic Obstr Pulm Dis* 2009; **6**: 446–51.

24 Primary care audit (Wales) 2015–17: Planning for every breath. London, 2017 https://www.rcplondon.ac.uk/projects/outputs/primary-care-audit-wales-2015-17-planning-every-breath (accessed Jan 19, 2018).

25 Mehuys E, Boussery K, Adriaens E, *et al.* COPD Management in Primary Care: An Observational, Community Pharmacy–Based Study. *Ann Pharmacother* 2010; **44**: 257–66.

26 Davis KJ, Landis SH, Oh Y-M, *et al.* Continuing to Confront COPD International Physician Survey: physician knowledge and application of COPD management guidelines in 12 countries. *Int J Chron Obstruct Pulmon Dis* 2015; **10**: 39–55.

27 Siddharthan T, Pollard SL, Quaderi SA, *et al.* Effectiveness-implementation of COPD case finding and self-management action plans in low- and middle-income countries: global excellence in COPD outcomes (GECo) study protocol. *Trials* 2018; **19**: 571.

28 Quality and Outcomes Framework Indicators. https://www.nice.org.uk/standards-and-indicators/qofindicators (accessed Jan 21, 2018).

For peer review only



For peer review only - http://bmjopen.bmj.com/site/about/guidelines.xhtml

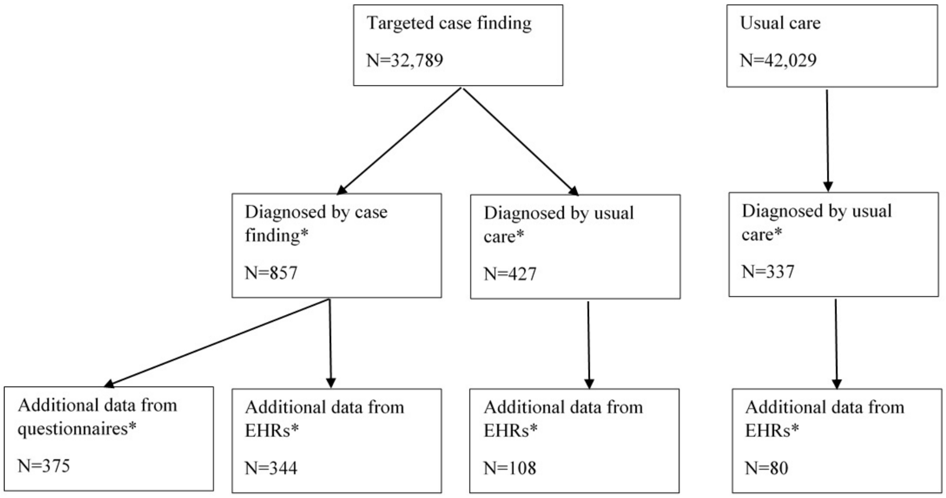


Figure 1 Participant flow diagram (*included in the current study; EHR=electronic health record)

203x114mm (144 x 144 DPI)

*Supplementary Table 1 Clinical codes used to extract data on COPD-related care from GP electronic health records*

| **Variable** | **Variable (specific)** | **Read code v2** | **Read code v3** |
|---|---|---|---|
| Antibiotic rescue pack | | "8IEZ","8BMW" | "XaW9D","XaZp7" |
| Anxiety screening | | "6897","388N" | "Xab9E","XaIwe" |
| Body mass index | | "22K%" | "22K%" |
| COPD Care plan | | "8CMV","66YI" | "8CMV","XaIUt" |
| Chest x-ray | | "536" | "XE2az" |
| Clinical COPD Questionnaire (CCQ) | | "38Dd" | "XaR6n" |
| COPD Assessment Test (CAT) | | "38Dg" | "XaRFy" |
| COPD exception reporting | | "9h5%" | "XaJ4R","XaJ4l","XaJ4k" |
| COPD review | | "66YM" | "XaIet","XaXCb","XaXCa" |
| COPD severity | | "H39","H36","H37","H38" | "XaEIV","XaN4a","XaEIY","XaEIW" |
| Depression screening | | "6896%","6891%","388P","388w","E204","3885","38G5","388f" | "6891%","XaIwf","XaNkT","62T1","XaYS4","XaLDN" |
| Influenza vaccine | | "65E%","n47%","9OX5" | "65E%","n47%","XaIBI" |
| Inhaler technique assessed/status | | "6636","663I","663H","6637","66Y4" | "6636","663I","663H","6637","XaIQ0" |
| Prednisolone | | "fe6%" | "fe6%" |
| Beclometasone dipropionate | | "c61%","c66%" | "c61%","c66%" |
| Budesonide | | "c64%" | "c64%" |
| Budesonide + formoterol (Symbicort) | | "c67%" | "c67%" |
| Fluticasone + salmeterol (Seretide) | | "c1D%" | "c1D%" |
| Fluticasone propionate | | "c65%" | "c65%" |
| Indacaterol | | "c1b%" | "c1b%" |
| Ipratropium bromide | | "c31%" | "c31%" |
| Salbutamol | | "c13%","c1E%" | "c13%","c1E%" |
| Salmeterol | | "c19%" | "c19%" |
| Terbutaline | | "c144","C145","C146","C147","c148","c14d","c14f","c14g","c14i","c14j","c14k","c14t","c14u","c14v","c14w","c14x" | "c144","C145","C146","C147","c148","c14d","c14f","c14g","c14i","c14j","c14k","c14t","c14u","c14v","c14w","c14x" |
| Aminophylline | | "c41%" | "c41%" |
| Theophylline | | "c43%" | "c43%" |
| MRC dyspnoea score | | "173H","173I","173J","173K","173L" | "XaIUl","XaIUm","XaIUn","XaIUo","XaIUi" |
| Oxygen saturation | | "44YA" | "X7708" |
| Oxygen therapy | | "8771%" | "8771%","X71a%","XALL8" |
| Palliative care patients | | "9EB5","8CM1%" | "XaJv2","XE2XB","XaCDx" |

For peer review only

| Variable | Variable (specific) | Read code v2 | Read code v3 |
|---|---|---|---|
| Physical activity level (GPPAQ) | | "138b","138X","138a","138Y" | "XaPPE","XaPP8","XaPPD","XaPPB" |
| Pneumococcal vaccine | | "6572","n4b%","8I3Q" | "6572","n4b%","XaIyy" |
| Pulmonary rehabilitation | | "8H7u","8FA%","9NSL","8I86","8IA9" | "XaIf9","XaIUp","XaIUq","XS7qP","XaIUs" ,"XaZuM","XabGM","XaNQU" |
| Smoking cessation - medication | Bupropion / Nicotine replacement therapy / Varenicline | "du6%", "du3%","du7%", "du8%" | "du6%", "du3%","du7%", "du8%" |
| Smoking cessation | Declined smoking cessation support | "9NdZ" | "XaRFh" |
| Smoking cessation | Smoking cessation advice | "8CAL" | "Ua1Nz" |
| Smoking status | Current/ex/never | "137%" | "137%" |
| Spirometry | FEV1: actual / predicted / % predicted, FEV1/FVC ratio: actual / predicted / % predicted, FVC: actual / predicted / % predicted | "339S","339O","339P","339R","339M","339 N","3396%","339Q" | "XaEFz","X77Qu","XaCJK","XaEFy","X77 Ra","XaCFR","3396%","XaQmT","XaPpI"," XaCJL","XaJ3K" |
| Spirometry | | "745D4","8HRC" | "XaXeg","XaK02" |

For peer review only

*Supplementary Table 2 Clinical codes used to define a clinician diagnosis of COPD*

| Clinical system: EMIS Web and LV | |
|---|---|
| H31 | Chronic bronchitis |
| H32 | Emphysema |
| H36 | Mild chronic obstructive pulmonary disease |
| H37 | Moderate chronic obstructive pulmonary disease |
| H38 | Severe chronic obstructive pulmonary disease |
| H39 | Very severe chronic obstructive pulmonary disease |
| H3A | End stage chronic obstructive airways disease |
| H3y | Other specific chronic obstructive airways disease |
| H3z | Chronic obstructive airways disease |
| Clinical system: System One | |
| H3 | COPD read codes in a cluster |

For peer review only

*Supplementary Table 3 Participant characteristics of those with and without additional electronic health record (EHR) or questionnaire data*

| **Participant characteristics** | **With additional EHR data** | **With follow-up questionnaire data** | **Without additional EHR or follow-up questionnaire data** |
|---|---|---|---|
| Mean age (SD) | 63.8 (9.4) | 64.7 (8.9) | 63.2 (10.2) |
| % Male | 62.2 | 58.7 | 54.2 |
| % Current smoker | 37.7 | 29.2 | 37.3 |
| Mean $FEV_1$ % predicted (SD) | 86.3 (18.1) | 87.8 (17.9) | 84.8 (18.4) |

*Supplementary Table 4 Participant characteristics of those with questionnaire data (n=375)*

| **Participant characteristics** | | **N** | **(%)** |
|---|---|---|---|
| Median age (IQR) | | 65.8 | (58.8-71.0) |
| Sex | Male | 220 | (58.7) |
| Smoking status | Current | 77 | (20.5) |
| | Former | 202 | (53.9) |
| | Never | 41 | (10.9) |
| $FEV_1$ % predicted | Mean | 87.8 | (17.9) |
| | ≥80 | 249 | (66.4) |
| | 50-79 | 121 | (32.3) |
| | <50 | 5 | (1.3) |
| Comorbidities | Asthma | 31 | (8.3) |
| | Ischaemic heart disease | 22 | (5.9) |
| | Diabetes | 20 | (5.3) |
| | Depression | 5 | (1.3) |
| | Anxiety | 6 | (1.6) |
| CAT score (median, IQR) | | 12 | (7-19) |
| Symptoms | MRC dyspnoea ≥3 | 148 | (39.5) |
| | Chronic cough | 128 | (34.1) |
| | Phlegm | 194 | (51.7) |
| | Wheeze | 274 | (73.1) |

CAT=COPD Assessment Test, FEV1=forced expiratory volume in 1 second

For peer review only

*Supplementary Table 5 Clinical care* of participants with electronic health record data who were newly diagnosed with COPD by case-finding and usual care*

| | | Diagnostic approach | | | |
|---|---|---|---|---|---|
| | | Case finding (n=341) | | Usual care (n=188) | |
| | | **N**** | **(%)** | **N**** | **(%)** |
| ***Clinical assessment*** | | | | | |
| MRC score recorded | | 107 | (31.4) | 176 | (93.6) |
| CAT score recorded | | 39 | (11.4) | 102 | (54.3) |
| Spirometry undertaken | | 101 | (29.6) | 147 | (78.2) |
| COPD severity recorded | | 48 | (14.1) | 118 | (62.8) |
| BMI recorded | | 277 | (81.2) | 178 | (94.7) |
| Oxygen saturations recorded | | 50 | (14.7) | 66 | (35.1) |
| CXR undertaken | | 22 | (6.5) | 20 | (10.6) |
| Depression screen undertaken | | 71 | (20.8) | 64 | (34.0) |
| ***Clinical intervention*** | | | | | |
| Listed on COPD register | | 78 | (22.9) | 175 | (93.1) |
| Care plan recorded | | 41 | (12.0) | 119 | (63.3) |
| Annual review undertaken | | 95 | (27.9) | 176 | (93.6) |
| Smoking cessation counselling provided | | 185 | (54.3) | 154 | (81.9) |
| Nicotine replacement therapy | | 39 | (11.4) | 29 | (15.4) |
| Influenza vaccination provided | | 245 | (71.8) | 144 | (76.6) |
| Pneumococcal vaccine provided | | 29 | (8.5) | 28 | (14.9) |
| Pulmonary rehabilitation provided | | 20 | (5.9) | 52 | (27.7) |
| Inhaler technique assessed | | 73 | (21.4) | 140 | (74.5) |
| Inhalers prescribed | Salbutamol | 146 | (42.8) | 167 | (88.8) |
| | Ipratropium | 8 | (2.3) | 15 | (8.0) |
| | Salmeterol | 3 | (0.9) | 15 | (8.0) |
| | Fluticasone | 3 | (0.9) | 1 | (0.5) |
| | Budesonide | 1 | (0.3) | 0 | (0.0) |
| | Beclometasone | 26 | (7.6) | 23 | (12.2) |
| | Fluticasone/salmeterol | 37 | (10.9) | 77 | (41.0) |
| | Budesonide/formoterol | 12 | (3.5) | 26 | (13.8) |
| | Any of the above inhalers | 151 | (44.3) | 172 | (91.5) |
| Antibiotic rescue pack | | 11 | (3.2) | 48 | (25.5) |
| Prednisolone | | 69 | (20.2) | 114 | (60.6) |
| **Clinical care score** | <5 | 225 | (66.0) | 17 | (9.0) |
| | 5 to 9 | 83 | (24.3) | 73 | (38.8) |
| | ≥10 | 33 | (9.7) | 98 | (52.1) |
| | Median (IQR) | 4 | (2 to 6) | 11 | (9 to 13) |

*Includes data from the start up to 2 years following the end of the TargetCOPD trial
**Number of participants who received the clinical assessment or intervention
BMI=body mass index, CAT=COPD Assessment Test, IQR=interquartile range, MRC=Medical Research Council

For peer review only

*Supplementary Table 6 Multilevel linear regression model assessing the association between the clinical care score* and participant characteristics, among those with electronic health record data (n=467)*

| **Participant characteristics** | **aβ*** | **(95% CI)** | **p** |
|---|---|---|---|
| Age | 0.03 | (0.00, 0.05) | 0.022 |
| Sex (male) | -0.35 | (-0.80, 0.09) | 0.121 |
| Smoking status | | | |
| Ex-smoker | 0.01 | (-0.84, 0.86) | 0.987 |
| Current smoker | 1.09 | (0.21, 1.97) | 0.015 |
| No. of comorbidities | 0.32 | (0.09, 0.55) | 0.006 |
| Case-found vs. routinely diagnosed | -0.48 | (-1.21, 0.24) | 0.189 |
| Listed on COPD register | 6.15 | (5.49, 6.82) | <0.001 |

* Includes data from the start up to 2 years following the end of the TargetCOPD trial

For peer review only

**STROBE 2007 (v4) checklist of items to be included in reports of observational studies in epidemiology***
**Checklist for cohort, case-control, and cross-sectional studies (combined)**

| Section/Topic | Item # | Recommendation | Reported on page # |
|---|---|---|---|
| **Title and abstract** | 1 | (*a*) Indicate the study's design with a commonly used term in the title or the abstract | 1 |
| | | (*b*) Provide in the abstract an informative and balanced summary of what was done and what was found | 2 |
| **Introduction** | | | |
| Background/rationale | 2 | Explain the scientific background and rationale for the investigation being reported | 5 |
| Objectives | 3 | State specific objectives, including any pre-specified hypotheses | 5, 6 |
| **Methods** | | | |
| Study design | 4 | Present key elements of study design early in the paper | 7 |
| Setting | 5 | Describe the setting, locations, and relevant dates, including periods of recruitment, exposure, follow-up, and data collection | 8 |
| Participants | 6 | (*a*) *Cohort study*—Give the eligibility criteria, and the sources and methods of selection of participants. Describe methods of follow-up<br>*Case-control study*—Give the eligibility criteria, and the sources and methods of case ascertainment and control selection. Give the rationale for the choice of cases and controls<br>*Cross-sectional study*—Give the eligibility criteria, and the sources and methods of selection of participants | 8 |
| | | (*b*) *Cohort study*—For matched studies, give matching criteria and number of exposed and unexposed<br>*Case-control study*—For matched studies, give matching criteria and the number of controls per case | N/A |
| Variables | 7 | Clearly define all outcomes, exposures, predictors, potential confounders, and effect modifiers. Give diagnostic criteria, if applicable | 8, 9 |
| Data sources/ measurement | 8* | For each variable of interest, give sources of data and details of methods of assessment (measurement). Describe comparability of assessment methods if there is more than one group | 8, 9 |
| Bias | 9 | Describe any efforts to address potential sources of bias | 9 |
| Study size | 10 | Explain how the study size was arrived at | 7, 8 |
| Quantitative variables | 11 | Explain how quantitative variables were handled in the analyses. If applicable, describe which groupings were chosen and why | 9 |
| Statistical methods | 12 | (*a*) Describe all statistical methods, including those used to control for confounding | 9 |
| | | (*b*) Describe any methods used to examine subgroups and interactions | N/A |
| | | (*c*) Explain how missing data were addressed | 9 |
| | | (*d*) *Cohort study*—If applicable, explain how loss to follow-up was addressed<br>*Case-control study*—If applicable, explain how matching of cases and controls was addressed | N/A |

For peer review only

| | | | |
|---|---|---|---|
| | | *Cross-sectional study*—If applicable, describe analytical methods taking account of sampling strategy | |
| | | (*e*) Describe any sensitivity analyses | 9 |
| **Results** | | | |
| Participants | 13* | (a) Report numbers of individuals at each stage of study—eg numbers potentially eligible, examined for eligibility, confirmed eligible, included in the study, completing follow-up, and analysed | 11 |
| | | (b) Give reasons for non-participation at each stage | N/A |
| | | (c) Consider use of a flow diagram | 20 |
| Descriptive data | 14* | (a) Give characteristics of study participants (eg demographic, clinical, social) and information on exposures and potential confounders | 11 |
| | | (b) Indicate number of participants with missing data for each variable of interest | 21 |
| | | (c) *Cohort study*—Summarise follow-up time (eg, average and total amount) | 7 |
| Outcome data | 15* | *Cohort study*—Report numbers of outcome events or summary measures over time | 11 |
| | | *Case-control study*—Report numbers in each exposure category, or summary measures of exposure | N/A |
| | | *Cross-sectional study*—Report numbers of outcome events or summary measures | N/A |
| Main results | 16 | (*a*) Give unadjusted estimates and, if applicable, confounder-adjusted estimates and their precision (eg, 95% confidence interval). Make clear which confounders were adjusted for and why they were included | 12, 13, 22, 25 |
| | | (*b*) Report category boundaries when continuous variables were categorized | N/A |
| | | (*c*) If relevant, consider translating estimates of relative risk into absolute risk for a meaningful time period | N/A |
| Other analyses | 17 | Report other analyses done—eg analyses of subgroups and interactions, and sensitivity analyses | 13 |
| **Discussion** | | | |
| Key results | 18 | Summarise key results with reference to study objectives | 14 |
| Limitations | 19 | Discuss limitations of the study, taking into account sources of potential bias or imprecision. Discuss both direction and magnitude of any potential bias | 16 |
| Interpretation | 20 | Give a cautious overall interpretation of results considering objectives, limitations, multiplicity of analyses, results from similar studies, and other relevant evidence | 17 |
| Generalisability | 21 | Discuss the generalisability (external validity) of the study results | 17 |
| **Other information** | | | |
| Funding | 22 | Give the source of funding and the role of the funders for the present study and, if applicable, for the original study on which the present article is based | 19 |

*Give information separately for cases and controls in case-control studies and, if applicable, for exposed and unexposed groups in cohort and cross-sectional studies.

**Note:** An Explanation and Elaboration article discusses each checklist item and gives methodological background and published examples of transparent reporting. The STROBE checklist is best used in conjunction with this article (freely available on the Web sites of PLoS Medicine at http://www.plosmedicine.org/, Annals of Internal Medicine at http://www.annals.org/, and Epidemiology at http://www.epidem.com/). Information on the STROBE Initiative is available at www.strobe-statement.org.

For peer review only
